# Prevalence and Determinants of Health Care Utilization Among Dutch Women in the First Year Postpartum

**DOI:** 10.1111/jmwh.70055

**Published:** 2025-12-04

**Authors:** Annemarie de Jong‐Bardelmeijer, Janneke Gitsels, Corine Verhoeven, Esther Feijen‐de Jong

**Affiliations:** ^1^ Department of Primary and Long‐Term Care University Medical Center Groningen University of Groningen Groningen the Netherlands; ^2^ Academy Midwifery Amsterdam and Groningen, Inholland Amsterdam the Netherlands; ^3^ Department of Midwifery Science Amsterdam UMC, Vrije Universiteit Amsterdam, Vrije Universiteit UMC Amsterdam the Netherlands; ^4^ Department of Quality of Care Amsterdam Public Health Research Institute Amsterdam the Netherlands; ^5^ Department of Obstetrics and Gynaecology Maxima Medical Center Veldhoven the Netherlands; ^6^ Division of Midwifery School of Health Sciences University of Nottingham Nottingham United Kingdom

**Keywords:** health services accessibility, health care utilization, maternal health services, maternal health, postnatal care, postpartum period

## Abstract

**Introduction:**

The first year after childbirth can be affected by persistent or new‐onset maternal health problems related to pregnancy and childbirth, leading to a reduction in quality of life, maternal well‐being, and mother‐child bonding. Evidence on determinants of health care utilization among postpartum women is limited by generalizability or the focus on 6 weeks postpartum. This study aimed to examine the prevalence of maternal health problems and health care utilization and to identify the determinants of health care utilization among postpartum women in the Netherlands within the first year after childbirth.

**Methods:**

A cross‐sectional design was used. Data were collected using a questionnaire from women 12 to 30 months postpartum in the Netherlands between March and May 2024. Health care utilization was measured as a proxy for health services. Nineteen potential determinants were derived from Andersen's behavioral model of health care utilization, of which 7 aligned with Levesque's complementary model of access to care and were analyzed using forward logistic regression.

**Results:**

In the sample of 1268 responses, 89.6% reported health problems, and 94.2% utilized health care in the first year after childbirth. The most frequently reported health problem was (extreme) fatigue (51.4%). The most common health care provider consulted was a general practitioner (73.2%). The odds of health care utilization were lower when postpartum women were unaware of health care services (odds ratio, 0.40; 95% CI, 0.25‐0.67).

**Discussion:**

The study demonstrated a high prevalence of health problems and health care use among Dutch postpartum women. Lack of awareness about the availability of health care was a key finding. Improving the dissemination of information about postpartum health care options may increase awareness of health problems beyond the immediate postpartum period. Additionally, findings emphasize the importance of long‐term counseling in addressing potential health problems.

## INTRODUCTION

The term *hidden morbidity*, as defined by Albers, refers to the period up to one year postpartum during which maternal health problems do not abate but rather persist or even emerge, as studies have shown.^1^, ^2^, ^3^, ^4^ A systematic review of 25 international studies identified a total of 83 maternal health problems in the first year after childbirth (postpartum), including extreme fatigue (48%) and back pain (43%) at 6 months and dyspareunia (21.4%), hemorrhoids (11.8%), and urinary incontinence (UI) (10.5%) at 12 months.^2^ The prevalence of pelvic‐girdle pain in Dutch postpartum women increased from 29% at 6 months to 35% at 12 months.^5^ These health problems associated with pregnancy and childbirth have a significant impact on quality of life, maternal well‐being, and mother‐child bonding.^6^, ^7^, ^8^, ^9^, ^10^, ^11^ There is a knowledge gap regarding the cost of and access to health care for such problems. However, maternal mental health problems, when considered independently, exert a significant economic burden and result in increased health care utilization in high‐income countries such as the United Kingdom, Canada, Australia, and the United States.^12^, ^13^ Improving continuity and responsiveness in postpartum care has been identified as a priority need by both academic literature and international quality improvement initiatives, providing opportunities for improvement in midwifery care.^4^, ^14^, ^15^

1 **QUICK POINTS**

✦ Understanding Dutch women's health problems after childbirth, women's use of health care after childbirth, and its determinants provides insights for improving access to health care.
✦ To facilitate access to postpartum health care, it is essential to prioritize women's ability to perceive and seek care and to develop interventions that address specific gaps.
✦ A 6‐week follow‐up is a valuable opportunity for maternal health care providers to increase awareness and ensure timely and appropriate care.
✦ It is essential to promote openness about postpartum health issues and the gap between awareness and access to health care.

Health care utilization is often used as a proxy to assess the performance, demand, and availability of health services.^16^ International findings on the overall prevalence of postpartum health care utilization are limited and often not applicable to the Dutch context, due to distinctive features of the Dutch health care system, such as standardized at‐home maternity care and mandatory health insurance. Existing data predominantly focus on specific health problems—such as pelvic pain and traumatic birth experience—or are limited to particular branches of health care, including contraceptive services, physiotherapy, or alternative medicine.^5^, ^11^, ^17^, ^18^, ^19^ The narrow focus presents a key limitation for studying overall health care utilization. In addition, analyses of overall health care utilization face limitations in cross‐national generalizability, primarily due to structural disparities between national insurance systems.^20^

Health care utilization is one of the steps in the sequence of access to care in Levesque's model. The model's approach provides a structured framework for assessing various aspects of access to care, with a particular focus on the processes that lead to health care utilization.^16^ The sequence of access to health care is influenced by the interaction of demand and supply‐side factors, which are shaped by the population and health system context.^16^ The supply side of the Levesque model addresses the characteristics and responsiveness of health care services and systems, focusing on how organizational and provider‐level factors facilitate or hinder access to care.^16^ The demand side of the Levesque model focuses on the client's perspective on access to health care and can be used to measure factors that influence usage.^16^ Complementary to the Levesque model, research has used Andersen's conceptual behavioral model of health care utilization to gain a deeper understanding of the factors influencing health care utilization.^21^ The Andersen model explains why and how health services are used and identifies determinants associated with utilization. It is presented in 4 components: predisposing, enabling, needing, and health behavior variables.^21^

Evidence on the determinants of health care utilization among postpartum women is limited. Similar to the evidence on prevalence, studies tend to cover periods shorter than one year postpartum, focus on specific health problems, or are conducted among subpopulations—such as socially disadvantaged or low‐income women—that are not representative of the broader postpartum population.^11^, ^18^, ^22^, ^23^, ^24^, ^25^

In the absence of Dutch prevalence rates of health problems and health care utilization, as well as generalizable determinants of postpartum women's use of health care services, we took the first step in measuring Dutch postpartum health care utilization, intending to enable health services to optimize the health and well‐being of women and children.^1^, ^6^, ^7^, ^8^ The Levesque and Andersen models reflect universal processes of access and utilization, enabling cross‐system comparison and contextual understanding.

The study provides a comprehensive overview of health care utilization among Dutch postpartum women in the first 12 months, within the framework of Andersen's model and Levesque's model, with a particular focus on the demand side of access to health care.^16^, ^21^ This study aimed to investigate 3 key areas: (1) the prevalence of self‐reported health problems among women in the period 6 weeks to 12 months postpartum in the Netherlands, (2) the prevalence of health care utilization among women in the Netherlands in the period 6 weeks to 12 months postpartum, and (3) the determinants of health care utilization of women in the first 12 months postpartum in the Netherlands.

## METHODS

### Participants

To be eligible for inclusion in the study, respondents had to be women aged 18 years or older, 12 to 30 months postpartum in the Netherlands, and have provided informed consent. Exclusion criteria included individuals who were not residents of the Netherlands, as well as women who were unable to read Dutch or English.

### Study Design and Setting

This study used a cross‐sectional, questionnaire‐based research design. Respondents were recruited between March 20 and May 10, 2024, through a collaboration with the Midwifery Academy Amsterdam‐Groningen and the University Medical Centers of Groningen and Amsterdam. A digital advertisement created by the Childbirth Network was disseminated through flyers at 201 municipal health departments (see Supporting Information: Figure S1).^26^ The advertisement was further disseminated via digital platforms associated with midwifery practices, obstetric collaborations and departments, patient organizations, maternal advisory councils, community‐based mother cafés, and midwives engaged in book publishing and blogging. The advertisement included a request for voluntary participation in an anonymous online questionnaire. Participants did not receive any form of incentive.

### Measurements

All measures were derived from the questionnaire, available in both Dutch and English, which contained both open‐ended and closed‐ended questions (Supporting Information: Appendices S1 and S2). Measures assessing sociodemographics, health care utilization, and health problems were adapted from the first (Q1) and third (Q3) questionnaires of the DELIVER study—a large national research project and modified to reflect the first postpartum year.^27^ Remaining measures were validated questions or developed specifically for this study, informed by existing literature and expert consultation, and aligned with the conceptual frameworks of Andersen and Levesque.^16^, ^21^ Efforts to reduce bias included refining the questionnaires after a pilot study with 10 participants.^28^ Feedback was gathered on linguistics, usability, readability, and recall time; however, only linguistic adjustments were necessary.

#### Health Problems

The prevalence of postpartum health problems was measured by asking about the occurrence of any of a predefined list of problems within 6 weeks to one year after childbirth. The list of health problems was based on the highest prevalence of postpartum health problems reported in the literature and included (extreme) fatigue; UI; hemorrhoids; back pain; pelvic pain; dyspareunia; stress, anxiety, or posttraumatic stress disorder (PTSD) due to (traumatic) childbirth experiences; low mood, anxiety, glooming, or depression; and no problems.^2^, ^29^ Women who reported at least one health problem were defined as having a health problem and were asked whether the problem existed before the most recent birth and whether the problem had worsened, with a yes or no response. Responses were categorized into 3 subcategories: (1) worsened: symptoms that preexisted and worsened after childbirth, (2) preexisting: symptoms that preexisted but remained unchanged, and (3) postpartum only: symptoms that emerged only postpartum.

#### Health Care Utilization

The frequency of health care utilization was measured by asking about the number of contacts with various health care providers between 6 weeks and one year after childbirth. For each provider, women were asked to report the number of contacts in predefined categories (0, 1‐3, 4‐6, 7‐9, 10‐12, 13‐15, >15 contacts), adapted from the DELIVER study.^27^ Contact was operationalized to encompass both face‐to‐face visits and remote communication, including telephone calls and telehealth consultations. The total of 32 providers was divided into 4 groups within the questionnaire based on consensus among the researchers, including multiprofessional health care providers, mental health care providers, complementary and alternative medicine, and institutions, with a list of all providers shown in Supporting Information: Appendix S2. Each group included an open‐ended text box in addition to the closed‐ended questions. Health care utilization was defined as a dichotomous variable, with a value of *yes* assigned if women reported at least one consultation with a listed health care provider.

#### Background Variables

The number of months postpartum was measured to assess the distribution of postpartum duration among the respondents. To ensure a representative sample for the Netherlands, respondents were asked about their province of residence, which was divided into the divisions North, West, East, and South based on the Dutch classification of Statistics Netherlands.^30^

#### Conceptual Frameworks

**Figure 1:**
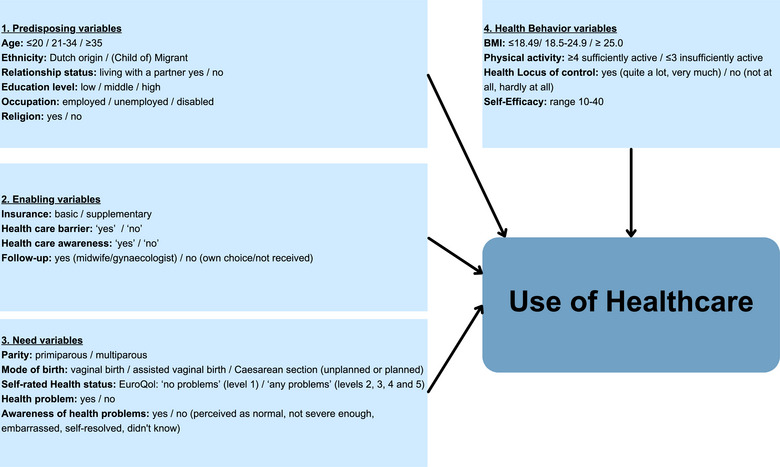
Andersen's Behavioral Model of Health Care Utilization: Framework and Operationalized Potential Determinants Abbreviations: BMI, body mass index; EuroQol: EQ‐5D‐5L.

**Figure 2:**
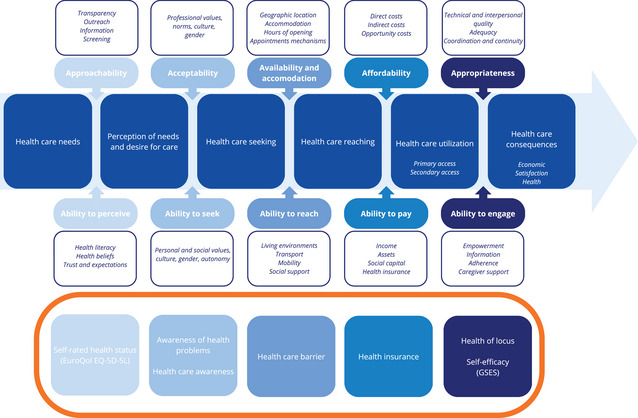
**Levesque's Conceptual Framework of Access to Health Care: Dimensions and Operationalized Demand‐Side Determinants**.^16^ Seven potential determinants from Andersen's Behavioral Model (shown in the surrounded box) are conceptually integrated into Levesque's Access to Care Framework Abbreviation: GSES, General Self‐Efficacy Scale.

To identify potential determinants, 2 conceptual and complementary frameworks were employed to explain health care utilization. The first, Andersen's model, is divided into 4 components potentially influencing health care utilization.^21^ Predisposing variables that influence health care utilization were mainly demographic, social, and belief‐related.^21^ Enablers, which were financial and organizational variables, can facilitate or act as barriers when absent.^21^ Perceived and valued health variables were categorized as need variables.^21^ Together with health behavior as a psychosocial variable, all influence the decision to use health care.^21^ Figure 1 illustrates the relationship between the components of Andersen's model and health care utilization, along with the distribution and operationalization of the study's potential determinants within the framework. The second, Levesque's model, integrates demand‐side ability dimensions with supply‐side accessibility dimensions to determine access.^16^ The present study focused on demand‐side ability dimensions to understand the client's perspective and identify factors influencing health care utilization. This approach helped identify areas for improvement in access to health care. Seven potential determinants from Andersen's model—self‐rated health status, awareness of health problems, health care awareness, health care barriers, health insurance, health locus of control, and self‐efficacy —were also applied to Levesque's model, as shown in Figure 2.

#### Potential Determinants

The rationale for each variable is described within the corresponding components of Andersen's model. Additionally, a separate subsection provides detailed information on the measurement details, following the same sequence.

#### Predisposing Variables

The following demographic variables were measured: age, relationship status, occupational status, level of education, religion, and ethnicity. Among younger women, both single status and living with a partner, unemployment, a low education level, religiosity, and ethnic minority status have been associated with lower health care utilization in the first 6 weeks postpartum in other populations.^22^, ^23^

#### Enabling Variables

The variable *health insurance*, for women who had no insurance, was found to act as a barrier to health care utilization in other populations.^23^ The variable health care barrier was used to measure the reasons why individuals encounter obstacles when attempting to access health care services, based on previous research and guided by the Levesque model. The variables of health care awareness and follow‐up due to a lack of knowledge have been identified as barriers to health care utilization.^23^ Health care awareness was measured to determine if and how women were aware of the availability of health care for the listed health problems.^23^ Follow‐up is part of standard care at 6 weeks postpartum; however, it is not used or offered to all postpartum women, yet it could lead to more referrals to other health care providers.

#### Need Variables

Mode of birth and parity have both been shown to influence health care utilization in other populations, with cesarean birth acting as a barrier, and conflicting evidence suggests that both being a multiparous woman and being a primiparous woman act as barriers to health care utilization.^23^ The variable self‐rated health status, as having a higher level of self‐rated health status, indicating poorer health status, was a reported determinant of higher health care utilization in a study of a specific health problem.^11^ Respondents’ self‐rated health status was measured using the EuroQol (EQ‐5D‐5L), with good test‐retest reliability (intraclass correlation coefficient [ICC] = 0.81). The variable awareness of health problems, as the ability of individuals to recognize and understand health problems as reasons to seek professional help, was measured because studies show that postpartum problems are not openly and commonly discussed due to cultural norms or women perceiving their problems as normal.^1^, ^23^

#### Health Behavior Variables

Measures of body mass index (BMI) and physical activity were based on clinical appropriateness as lifestyle measures and their association with increased health care use in the general population.^31^ Physical activity was quantified using the Dutch Physical Activity Questionnaire (VFA), which demonstrated good test‐retest reliability (ICC = 0.76).^32^ Empowerment, when low, has been shown to act as a barrier in other populations.^22^, ^33^ The concept of empowerment comprises 2 components: locus of control over one's health and self‐efficacy.^22^, ^33^ Self‐efficacy was measured using the General Self‐Efficacy Scale (GSES), which demonstrated good internal consistency (Cronbach α = 0.85).^34^

#### Measurement of Potential Determinants

All variables and corresponding measurement categories are shown in the Andersen model in Figure 1. An overview of all variable measurements is presented in the Supporting Information: Table S1.

#### Predisposing Variables

Age was measured continuously, and the data were categorized according to the classification of Statistics Netherlands.^35^ Ethnicity consists of an aggregated variable formed by combining responses (yes or no) from 3 questions: (1) Dutch origin: respondent and both parents born in the Netherlands, (2) child of migrant: respondent born in the Netherlands with 1 or 2 parents born abroad, and (3) migrant: foreign‐born respondent^36^. Subsequently, *child of migrant* and *migrant* were merged into one category, creating a dichotomous variable. Education level was categorized into 3 groups: low (primary education, prevocational secondary education, the first 3 years of senior general secondary education, or entry‐level vocational training); middle (upper years of senior general secondary education, or intermediate vocational training levels); and high (higher professional education, university education, or postacademic education).^37^, ^38^ Responses for occupational status were grouped into 3 categories: (1) employed, which included both students and individuals with paid employment; (2) unemployed, comprising those actively seeking work as well as homemakers; and (3) disabled, which included individuals who were either entirely or partially unfit for work. The category “Other, namely” was excluded from the analysis and grouped as missing data.

#### Enabling Variables

For health care barrier, previous research was used to select the response options, which were grouped for analysis into 2 categories: *yes* (personal reasons, proximity of health facility, lack of time, lack of awareness) and *no* (not needed, adequate care, concerns not severe enough, self‐resolving, inflexible opening hours, financial constraints), guided by the demand side of Levesque's model.^23^, ^39^ For health care awareness, responses were grouped into 2 categories: *yes* (through referral, through social media, through Google, through family or friends, through advertising) and *no* (no, not needed).

#### Need Variables

The EQ‐5D‐5L asks the respondent to consider and rate her current state of health in 5 dimensions: mobility, self‐care, usual activities, pain or discomfort, and anxiety or depression.^40^ Responses to questions on each dimension could take 1 of 5 values covering 5 levels of severity (no problems, slight problems, moderate problems, severe problems, extreme problems).^40^

#### Health Behavior Variables

BMI was calculated from the respondent's current weight and height using the Quetelet index and classified according to the World Health Organization's classification.^41^ The VFA comprises 2 questions about physically intensive activities (scored as 3 times per week, 4; 1‐2 times per week, 2; never, 0) and moderately intensive activities (scored as 5 times per week, 4; 3‐4 times per week, 2; 1‐2 times per week, 1; never, 0).^32^ The VFA score is the sum of both questions, with a range of 0 to 8, divided into sufficiently active (≥4) or insufficiently active (≤3).^32^ The GSES assessed how a person generally copes with stressors or difficult situations in life. Ten statements were rated on a 4‐point scale (completely disagree, 1; slightly agree, 2; somewhat agree, 3; and completely agree, 4).^34^ The GSES scale ranges from 10 to 40, with higher scores indicating greater self‐efficacy.^34^ The mean for the general Dutch population is 32.^42^

### Sample Size, Ethics, and Guidelines

The minimum required sample size was 1050, based on the formula n = 100 + 50 ⋅ *i*, where 100 is the fixed baseline, 50 reflects the recommended events‐per‐variable, and *i* denotes the number of independent variables (*i* = 19).^43^ The process of informed consent was initiated at the beginning of the questionnaire, which was available in both Dutch and English. Participants indicated their consent by selecting “Yes” to proceed. Ethical approval was granted on February 13, 2024, by the Medical Ethics Review Committee of the University Medical Center Groningen (reference number 18133). The study followed the Strengthening the Reporting of Observational Studies in Epidemiology guidelines for reporting study results (Supporting Information: Appendix S3).^44^

### Statistical Analyses

Normally distributed continuous variables were summarized as means (SD), nonnormally distributed variables as medians and interquartile ranges (IQRs), and categorical variables as frequencies and percentages. Group differences were tested using 2‐sample *t* tests for numerical variables and Fisher's exact tests for categorical variables. Frequencies of missing data were calculated, and patterns were explored using matrix plots. Multiple imputation was used for missing data when missing (completely) at random. Prevalence rates of health problems were presented as percentages per problem, including subcategories such as preexisting, worsened, and postpartum only, for each health problem. Prevalence rates of health care utilization were presented as percentages per problem and health care provider. To minimize the risk of overfitting, a forward selection procedure was used for inferential statistics. Forward selection ensures control over the analysis and allows for the evaluation of which variables entered the model at each step, providing insight into both the individual and combined effects. First, univariable logistic regression analyses were performed for all potential determinants, with the results presented as the crude odds ratio and 95% CI. Second, the variable with the lowest significance level was identified as the first variable in the main model. Subsequently, the remaining variables were added to the main model separately, with the selection process repeated and the main model built until all remaining variables failed to meet the selection criterion of *P* value <.05. The identified determinants were presented with adjusted odds ratios (aORs) and 95% CI. A sensitivity analysis was performed using the area under the curve (AUC), calculated from the receiver operating characteristic curve, which plots the frequency at which the model correctly identifies positive cases at various thresholds. The AUC assesses the model's ability to distinguish between individuals with and without the outcome. The values 0.5 and 1.0 correspond to *no* and *perfect discriminative performance*, respectively. The model based on complete case analysis was compared with the model using imputed data. Comparable results suggest that imputation had little impact on model performance. R‐studio (R 3.3.0+) was used for the analysis.

## RESULTS

### Participants Descriptive Statistics

A total of 1268 responses were received. The median time to complete the questionnaire was 10.72 minutes (IQR, 8.59‐14.03). Overall, there were limited missing data (1.1%). The highest percentage of missing data was for the follow‐up question (3.9%), which was the last question. The location of missing data is shown in the Supporting Information: Figure S2 flowchart. A total of 33 (2.6%) responses were missing for health care utilization, which is considered minimal when appropriately addressed.^45^, ^46^ The missing observations in the matrix plots were independent of other variables or outcomes in the data set, so multiple imputation was used. Respondent demographics, presented in Table 1, indicate that most women were of Dutch origin (86.4%), highly educated (73.9%), and with a median postpartum period of 19.0 months (IQR, 15.0‐24.0).

**Table 1:** Background Characteristics and Their Associations With Health Care Utilization in the First Postpartum Year (N = 1268)

	Health Care Utilization					
Background Characteristics	Total N = 1268	Yes 1163 (94.2%)	No 72 (5.8%)	*P* Value	Univariable Logistic Regression Analysis^a^ Crude OR (95% CI)	Forward Regression Analysis^b^ Adjusted OR (95% CI)
**Mo postpartum (range, 12‐30),** **median (IQR)**	19 (15‐24)	19 (15‐24)	20 (15‐25)	.68^c^	N/A	N/A
**Dutch region, n (%)**				.51^d^	N/A	N/A
North	153 (12.1)	145 (12.5)	7 (9.7)			
West	457(36.0)	412 (35.4)	29 (40.3)			
East	464 (36.6)	428 (36.8)	29 (40.3)			
South	194 (15.3)	178 (15.3)	7 (9.7)			
**Age, n (%), y**				.07^d^		
≤20^e^	1 (0.1)	0 (0)	1 (1.4)			
21‐34	1043 (82.2)	956 (82.2)	58 (80.6)		1 [Reference]	
≥35	224 (17.7)	207 (17.8)	13 (18.0)		0.97 (0.54‐1.88)	
**Ethnicity, n (%)**				.86^d^		
Dutch origin	1096 (86.4)	1008 (86.7)	62 (86.1)		1 [Reference]	
(Child of) migrant	172 (13.6)	155 (13.3)	10 (13.9)		0.97 (0.51‐2.05)	
**Relationship status, n (%)**				.15^d^		
Married, registered partnership, living together	1231 (97.1)	1131 (97.2)	68 (94.4)		1 [Reference]	
Living alone, no relationship	37 (2.9)	32 (2.8)	4 (5.6)		0.48 (0.17‐1.40)	
**Education level, n (%)**				.16^d^		
High	937 (73.9)	869 (74.7)	48 (66.6)		1 [Reference]	
Middle	302 (23.8)	271 (23.3)	21 (29.2)		0.72 (0.43‐1.25)	
Low	29 (2.3)	23 (2.0)	3 (4.2)		0.47 (0.16‐2.01)	
**Occupational status, n (%)**				.34^d^		
Employed	1174 (93.3)	1084 (93.4)	66 (93.0)		1 [Reference]	
Unemployed	59 (4.7)	52 (4.5)	5 (7.0)		0.79 (0.36‐2.09)	
Disabled^f^	25 (2.0)	25 (2.1)	0 (0)			
Missing^g^	10	8	1			
**Religious background, n (%)**				.002^d^		
No	998 (78.7)	927 (79.7)	45 (62.5)		1 [Reference]	
Yes	270 (21.3)	236 (20.3)	27 (37.5)		0.43 (0.26‐0.71)	0.40 (0.24‐0.68)

Abbreviations: IQR, interquartile range; N/A, not applicable for regression analysis.

a Logistic model assumptions: no multicollinearity (Variance Inflation Factor [VIF] < 10), errors were independent by Durbin‐Watson test (*dw* = 2.06, *P* =  .845), the event‐per‐variable criterion allowed 7 parameters, for linearity in the logit the log of GSES was statistically significant (β = 11.45, *z* = 3.84, *P* <  .001) and was transformed with the restricted cubic spline function for a better fit (β = ‐0.17; *Wald Z* = −2.89, *P* =  .004).

b The final forward regression model was adjusted for (1) health care awareness, (2) health insurance, (3) religion, (4) self‐rated health status, (5) awareness of health problems, (6) self‐efficacy.

c The assumptions of normal distribution and the homogeneity of variance were met, validating the use of a two‐sample *t* test.

d Analysis by Fisher's exact test.

e Category “≤20” merged with “21‐34” due to empty cells in the health care utilization group for logistic regression analysis.

f Category “Disabled” merged with “Unemployed” due to empty cells in the no health care utilization group for logistic regression analysis.

g The discrepancy in missing values for health care utilization subgroups is attributable to its placement in the final section of the questionnaire, which resulted in 33 missing values for that variable alone.

#### Prevalence of Health Problems

**Figure 3:**
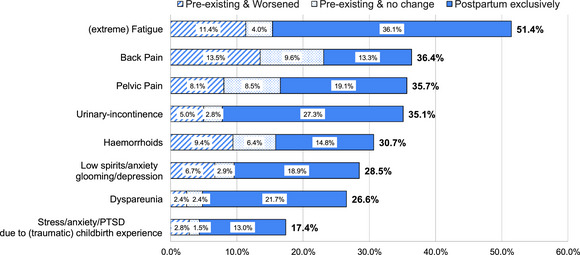
**Distribution of Self‐Reported Health Problems Among Women in the First 12 Months Postpartum in Percentages**. The subcategories of preexisting and worsened, preexisting and no change, and postpartum only, for each health problem in percentages Abbreviation: PTSD, posttraumatic stress disorder.

Overall, 89.6% of women reported at least one of the health problems listed, whereas 10.4% reported none of the health problems. The most frequently reported health problem was (extreme) fatigue (51.4%), followed by back pain (36.4%), as shown in Figure 3. The health problems reported in the open text box are shown in Supporting Information: Table S2.

#### Prevalence of Health Care Utilization

Overall, 94.2% of women reported at least one visit to a health care provider, whereas 5.8% reported not using any of the listed health care providers. Prevalences of health care providers visited are shown in Supporting Information: Figure S3. The health care providers reported in the open text box are listed in Supporting Information: Table S3.

### Inferential Statistical Results

The determinants of health care utilization among postpartum women in the first year are presented in Tables 1 and 2. Forward selection identified 6 determinants. The odds of health care utilization were lower for women with a religious background compared with women without a religious background (aOR, 0.40; 95% CI, 0.24‐0.68). In addition, the odds of health care utilization were lower for women who were unaware of the availability of health services for the listed health problems as opposed to women who were aware (aOR, 0.40; 95% CI, 0.25‐0.67). Furthermore, the odds of health care utilization decreased with each additional point on the GSES scale, which measures self‐efficacy (aOR, 0.88; 95% CI, 0.78‐0.98). In contrast, the odds of health care utilization were higher among women with supplementary health insurance compared with women with basic health insurance (aOR, 3.18; 95% CI, 1.92‐5.27). Women who reported “any problems” on self‐rated health status had higher odds of health care utilization compared with women who reported “no problems” (aOR, 2.28; 95% CI, 1.32‐3.93). In addition, the odds of health care utilization were higher among women who were aware of the possibility of contacting health care providers for the listed health problems compared with women who were unaware (aOR, 1.85; 95% CI, 1.04‐3.29). Sensitivity analysis showed almost identical model performance for complete case analysis (AUC 0.784) and imputed data analysis (AUC 0.783), with no significant difference according to the Delong test (*D* = −0.01, *P* =  .992).

**Table 2:** Maternal Health, Access, and Psychosocial Characteristics Associated With Health Care Utilization in the First Postpartum Year (N = 1268)

	Health Care Utilization					
Maternal Health, Access, and Psychosocial Characteristics, n (%)	Total N = 1268	Yes 1163 (94.2%)	No 72 (5.8%)	*P* Value	Univariable Logistic Regression Analysis^a^ Crude OR (95% CI)	Forward Regression Analysis^b^ Adjusted OR (95% CI)
**Health insurance, n (%)**				<.001^d^		
Basic	299 (23.8)	256 (22.2)	38 (53.5)		1 [Reference]	
Supplementary	958 (76.2)	897 (77.8)	33 (46.5)		3.97 (2.45‐6.45)	3.18 (1.92‐5.27)
Missing	11	10	1			
**Health care barrier, n (%)**				.51^d^		
No	1031 (84.0)	968 (83.8)	63 (87.5)		1 [Reference]	
Yes	196 (16.0)	187 (16.2)	9 (12.5)		1.38 (0.71‐3.02)	
Missing^e^	41	8	0			
**Health care awareness, n (%)**				<.001^d^		
Yes	985 (80.3)	946 (82.0)	39 (54.2)		1 [Reference]	
No	241 (19.7)	208 (18.0)	33 (45.8)		0.26 (0.16‐0.42)	0.40 (0.25‐0.67)
Missing^e^	42	9	0			
**Follow‐up, n (%)**				>.99^d^		
Yes	1079 (88.6)	1015 (88.6)	64 (88.9)		1 [Reference]	
No	139 (11.4)	131 (11.4)	8 (11.1)		1.05 (0.52‐2.42)	
Missing^e^	50	17	0			
**Parity, n (%)**				.11^d^		
Primiparous	716 (56.6)	661 (56.8)	34 (47.2)		1 [Reference]	
Multiparous	549 (43.4)	502 (43.2)	38 (52.8)		0.67 (0.41‐1.08)	
Missing^e^	3	0	0			
**Mode of birth, n (%)**				.10^d^		
Vaginal birth	909 (71.8)	831 (71.4)	59 (82.0)		1 [Reference]	
Assisted vaginal birth	116 (9.2)	108 (9.3)	6 (8.3)		1.27 (0.58‐3.35)	
Cesarean birth	240 (19.0)	224 (19.3)	7 (9.7)		2.30 (1.11‐5.60)	
Missing^e^	3	0	0			
**Self‐rated health status, n (%)**				<.001^d^		
No problems	473 (37.6)	416 (35.8)	47 (65.3)		1 [Reference]	
Any problems	785 (62.4)	747 (64.2)	25 (34.7)		3.35 (2.05‐5.60)	2.28 (1.32‐3.93)
Missing^e^	10	0	0			
**Health problems, n (%)**				.008^d^		
Yes	1113 (89.6)	1051 (90.4)	57 (79.2)		1 [Reference]	
No	129 (10.4)	112 (9.6)	15 (20.8)		0.41 (0.23‐0.78)	
Missing^e^	26	0	0			
**Awareness health problems, n (%)**				.02^d^		
No	234 (19.1)	212 (18.3)	22 (30.6)		1 [Reference]	
Yes	993 (80.9)	943 (81.7)	50 (69.4)		1.94 (1.13‐3.23)	1.85 (1.04‐3.29)
Missing^e^	41	8	0			
**BMI, n (%)**				.27^d^		
≤18.4	30 (2.4)	27 (2.3)	3 (4.2)		0.60 (0.20‐2.60)	
18.5‐24.9	588 (46.5)	537 (46.2)	37 (51.4)		1 [Reference]	
≥25.0	647 (51.1)	599 (51.5)	32 (44.4)		1.29 (0.79‐2.11)	
Missing^e^	3	0	0			
**Physical activity, n (%)**				.90^d^		
≥4 sufficient	659 (52.2)	609 (52.4)	37 (51.4)		1 [Reference]	
<3 insufficient	604 (47.8)	554 (47.6)	35 (48.6)		0.97 (0.60‐1.56)	
Missing^e^	5	0	0			
**Health locus of control, n (%)**				.27^d^		
Yes	1138 (91.6)	1062 (91.3)	69 (95.8)		1 [Reference]	
No	104 (8.4)	101 (8.7)	3 (4.2)		2.19 (0.80‐9.06)	
Missing^e^	26	0	0			
**Self‐efficacy, median (IQR)**				.25^c^		
Total score (10‐40)	33 (30.0‐36.0)	33 (30.0‐36.0)	35 (30.0‐37.0)		1.08 (0.99‐1.17)	1.06 (0.97‐1.16)
Missing^e^	26	0	0			
Total score (10‐40)^f^					0.85 (0.76‐0.95)	0.88 (0.78‐0.98)

Abbreviations: BMI, body mass index; IQR, interquartile range.

a Logistic model assumptions: no multicollinearity (Variance Inflation Factor [VIF] < 10), errors were independent by Durbin‐Watson test (*dw* = 2.06, *P* =  .845), the event‐per‐variable criterion allowed 7 parameters, for linearity in the logit the log of GSES was statistically significant (β = 11.45, *z* = 3.84, *P* <  .001) and was transformed with the restricted cubic spline function for a better fit (β = −0.17; *Wald Z* = −2.89, *P* =  .004).

b The final forward regression model was adjusted for (1) health care awareness, (2) health insurance, (3) religion, (4) self‐rated health status, (5) awareness of health problems, (6) self‐efficacy.

c The assumptions of normal distribution and the homogeneity of variance were met, validating the use of a two‐sample *t* test.

d Analysis by Fisher's exact test.

e The discrepancy in missing values for health care utilization subgroups is attributable to its placement in the final section of the questionnaire, which resulted in 33 missing values for that variable alone.

f Self‐efficacy transformed with the restricted cubic spline function for logistic regression analysis.

## DISCUSSION

This cross‐sectional study showed that almost 9 of 10 Dutch women experience at least one health problem in the first year after childbirth, the most common problems being (extreme) fatigue and back pain. Almost all postpartum women in the study have used health services, primarily general practitioners and physiotherapists. In terms of determinants, women were less likely to use health services in the first year after childbirth if they were unaware of available health services, had a religious background, or had higher self‐efficacy. Conversely, women were more likely to use health services if they had supplementary health insurance, had “any problems” according to their self‐rated health status, or were aware of the possibility of contacting health care providers for the listed health problems.

### Interpretation

#### Prevalence of Health Problems

For the most part, the proportion of women experiencing health problems aligns with international literature.^2^, ^3^ A discrepancy is observed in the prevalence of UI, with global estimates ranging from 10% to 60%.^2^, ^7^ The observed discrepancy likely stems from differences in self‐reporting measures and recall regarding overall prevalence within the first year postpartum. Moreover, considerable heterogeneity arises when various validated measurement instruments are employed across studies.^2^ While distinguishing between general distress and PTSD, this research measures all outcomes collectively, whether minor or major. Although PTSD accounts for an estimated 2% to 3% of cases, this study combines both subclinical symptoms and diagnosed disorders, resulting in overall mental health outcomes related to the childbirth experience that are consistent with previous research.^2^, ^29^

#### Prevalence of Health Care Utilization

Health care utilization in the first year postpartum varies significantly, with a notably high rate of physiotherapy. In the Netherlands, 6 in 10 women use physiotherapy, compared with 1 in 5 internationally.^5^ This is likely due to the high prevalence of back pain, pelvic pain, and UI, and the availability of self‐referral physiotherapy. In addition, coverage of physiotherapy through Dutch supplementary insurance may contribute to higher utilization rates, reflecting broader associations between health care use, insurance coverage, and income.^18^, ^25^ Additionally, frequent midwife consultations after 6 weeks may be linked to contraceptive care, which Dutch midwives have been authorized to prescribe since 2015. A comparison with existing literature was not possible, as available studies report prevalence rates of contraceptive use but do not specify the type of health care provider involved. Nevertheless, addressing women's reproductive health needs in the postpartum period is important, as these needs are frequently reported as unmet and face similar barriers in access to health care.^19^ In addition, compared with midwife consultations, nearly three‐quarters of the respondents reported contact with a general practitioner. This difference may reflect variations in perceived roles, accessibility, or awareness of midwifery services during the postpartum period.

#### Determinants Within the Conceptual Frameworks

Determinants were found in all 4 components of the Andersen model. The enabling component's determinants align with those identified in international research on subpopulations, including health insurance and health care awareness, which act as facilitators of personal skills and resources.^23^ Similarly, determinants in the need component, like lower self‐rated health and awareness of listed health problems, help to understand women's specific health needs and their impact on health care utilization.^23^ Identifying self‐efficacy in the health behavior component reveals its influence on women's decisions and behavior regarding the utilization of health services, a measurement that is frequently overlooked and found to be minimal in research.^22^, ^23^ Religion in the predisposing component is a rare determinant in previous research. The dichotomous nature of the religion question may oversimplify the complexity of religious beliefs and their interaction with cultural norms.^23^, ^47^ Additionally, cultural norms related to the acceptability dimension of Levesque's model influence the ability to seek and accept health care, potentially limiting access for women with a religious background.^16^ Levesque's model identifies determinants in the first 2 dimensions, highlighting areas for improvement to enhance access to health care. Key factors include increasing women's ability to perceive and seek health care, as postpartum women must be aware of services and health problems to access, pay for, and engage with them.

### Strengths and Limitations

A strength of the research is its contribution to the existing literature, being the first study in the Netherlands to examine health care utilization and health problems among women in the first year after childbirth, using a large sample and numerous potential determinants. The determinants included all 4 components of the Andersen model and all 5 dimensions of the Levesque model, providing a rationale for the findings in terms of explaining health care utilization. In terms of generalizability, the study sample proportions align with the Dutch population for most demographic variables.^30^, ^34^, ^36^, ^38^, ^48^ However, the higher proportion of women with a higher education level (73.9%) compared with the maternal Dutch population (57.0%) may limit generalizability due to the association of higher education level with higher health care utilization and may introduce self‐selection bias.^38^ The classification of ethnicity based on Dutch standards, along with the structure of the national insurance system, may limit the applicability and generalizability of the findings to health care systems outside the Netherlands. However, the use of conceptual models, such as those by Levesque and Andersen, which capture essentially universal processes related to health care access and utilization—such as awareness of health needs, self‐efficacy, and self‐rated health—enables meaningful comparison and interpretation across diverse health care systems. We acknowledge several limitations. Participants mainly responded through online advertising, increasing the likelihood of self‐selection and selection bias. There is a potential for recall bias, as questions on health care utilization and health problems were asked retrospectively. However, efforts to reduce recall bias included minimizing question detail, presetting responses, pretesting the questionnaire, and using validated questions where available.^28^ In addition, a shorter recall period is recommended when reporting visits to health care providers, as these are generally less salient events compared with hospitalizations.^49^ Although a one‐year recall period is considered appropriate for estimating average annual hospital use, our study allowed for a longer recall period.^49^ Nevertheless, the median recall duration of 19 months is at the upper limit of what is generally considered acceptable. For health problems, the study aimed to explore women's experiences rather than diagnose, recognizing the subjective nature of reported problems. It is acknowledged that self‐report questions may result in the overestimation or underestimation of health problem prevalence due to variations in awareness and recall among respondents. Moreover, the use of nonspecific terms such as *extreme fatigue* may reflect alternative etiologies beyond sleep deprivation, introducing potential unmeasured confounding.

### Implications

To facilitate access to postpartum health care, it is essential to prioritize women's ability to perceive and seek care, and to develop interventions that address specific gaps.^16^ Additional follow‐up care is a valuable opportunity for midwives and other maternal health care providers to increase awareness and ensure timely and appropriate care. Promoting openness about postpartum health problems and the discrepancy between awareness and access is crucial. In addition, early identification of barriers during pregnancy enables midwives to tailor care to individual needs and enhance continuity into the postpartum period.

We advocate for using social media, websites, and leaflets to disseminate information and promote awareness of postpartum health problems and health care options, supported by policies to ensure legitimacy, consistency, and sustainable impact. Addressing barriers, such as religion, through respectful dialogue that considers religious values, combined with efforts to improve access to supplementary health insurance, can reduce barriers for women. In addition, midwives may need to reassess the social support system or financial situation of families, particularly for women from low socioeconomic backgrounds. This reassessment should, in part, capture the complexity of access to health care across different global health systems through mixed methods, incorporating quality of care data, epidemiological surveys, and organizational surveys. To develop a comprehensive understanding of health care access by exploring the supply‐side aspects of the Levesque model, it is essential to incorporate the perspectives of health care providers and organizations, as the model's dimensions represent interrelated constructs.

## CONCLUSION

Disseminating information about health care options after childbirth raises awareness of postpartum health problems, highlights the need for long‐term guidance in the Dutch postpartum health care system, and emphasizes the importance of additional follow‐up care. Striving for a better quality of life for women will facilitate a more positive mother‐child bond, positively impacting the child's development.^6^, ^7^, ^8^, ^9^, ^10^ This research aims to improve maternal health services to provide more mother‐centered care, reduce health problems in the extended postpartum period, give women a better start to a new pregnancy, and ultimately improve quality of life, maternal well‐being, and mother‐child bonding.^1^, ^6^, ^7^, ^8^, ^9^, ^10^

## CONFLICT OF INTEREST

The authors have no conflicts of interest to disclose.

## Supporting information




**Appendix S1**. Dutch Questionnaire

**Appendix S2**. English Questionnaire

**Appendix S3**. Completed STROBE Checklist

**Figure S1**. Recruitment Advertising Online and Flyer

**Figure S2**. Flowchart of Missing Data in the Questionnaire

**Figure S3**. Distribution of Health Care Utilization Among Women in the Period 6 Weeks To 12 Months Postpartum, per Type of Health Care Provider, in Percentages

**Table S1**. Measurement and Categorization of Questionnaire Variables

**Table S2**. Distribution of Health Problems Reported by Respondents in the Open Text Box

**Table S3**. Distribution of Health Care Providers Reported by Respondents in the Open Text Box
