# Supplementary figures and images for: Prevalence and Determinants of Health Care Utilization Among Dutch Women in the First Year Postpartum
Source: J Midwifery Womens Health. 2025 Dec 4;71(1):113–25. doi: 10.1111/jmwh.70055 (PMC12914622; doi:10.1111/jmwh.70055)

# **
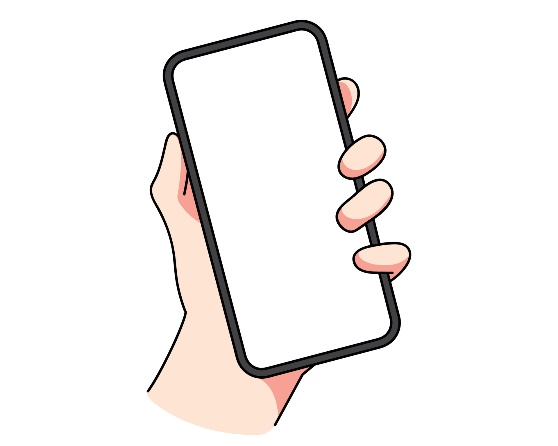
Supplemental Information** **- Questionnaire Dutch**


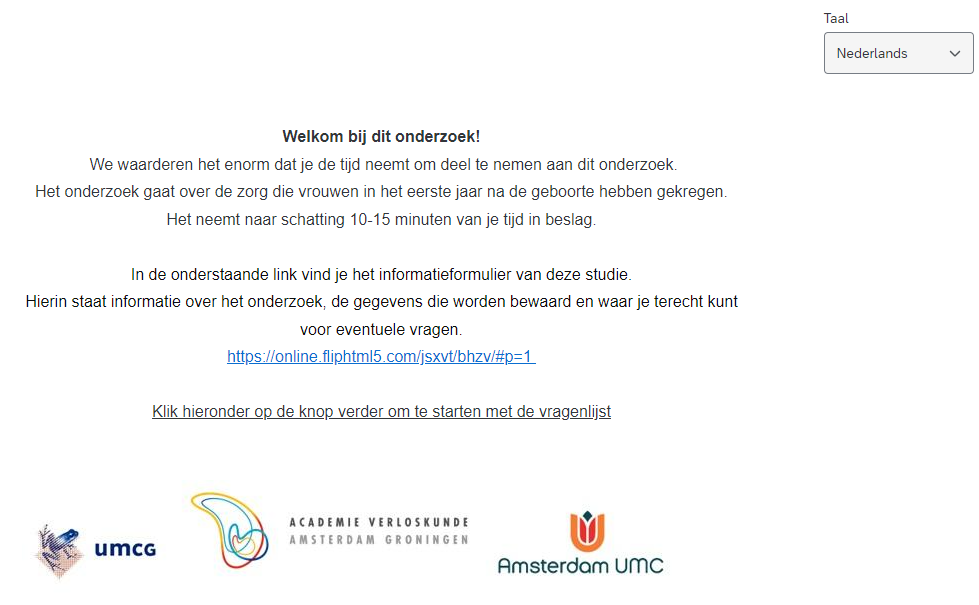


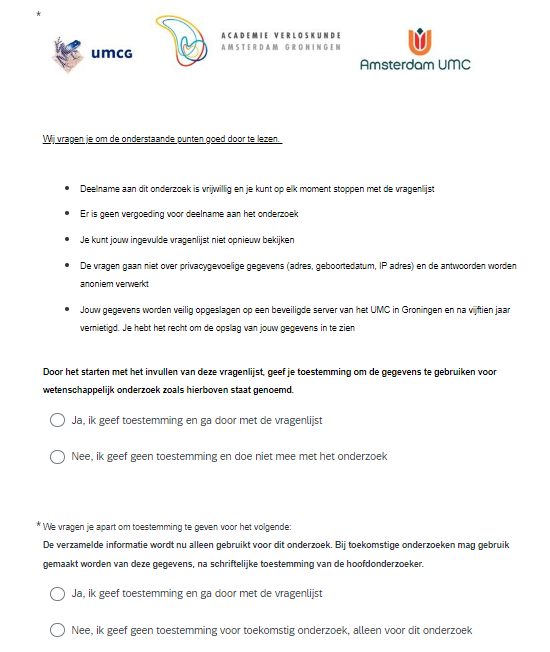


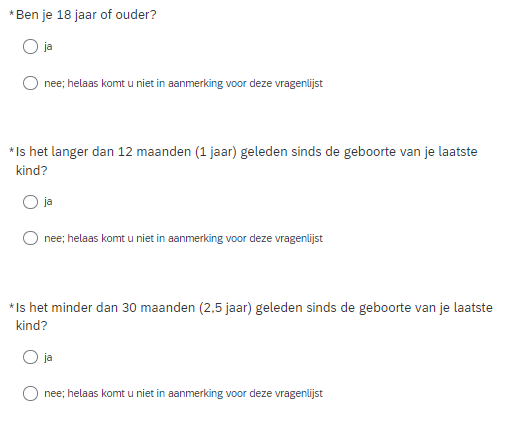


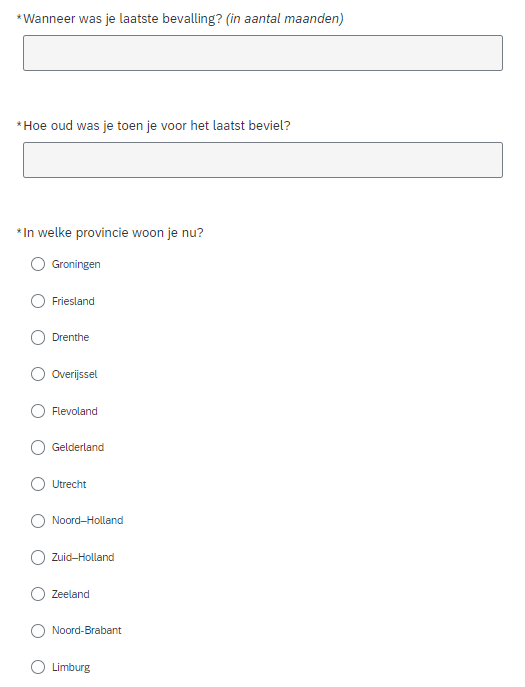


*
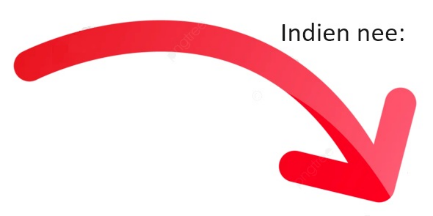

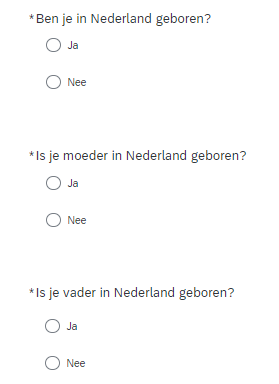

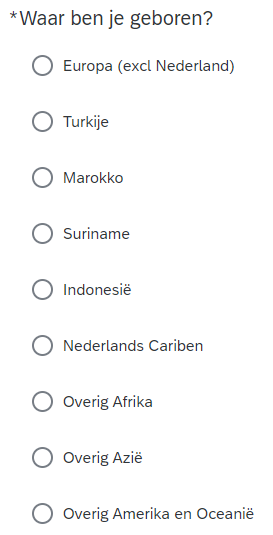
*
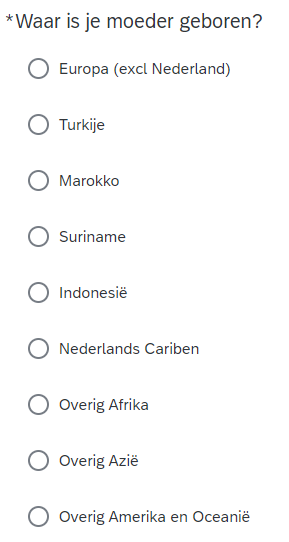
*
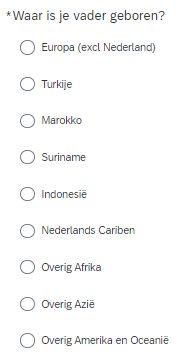
*

*
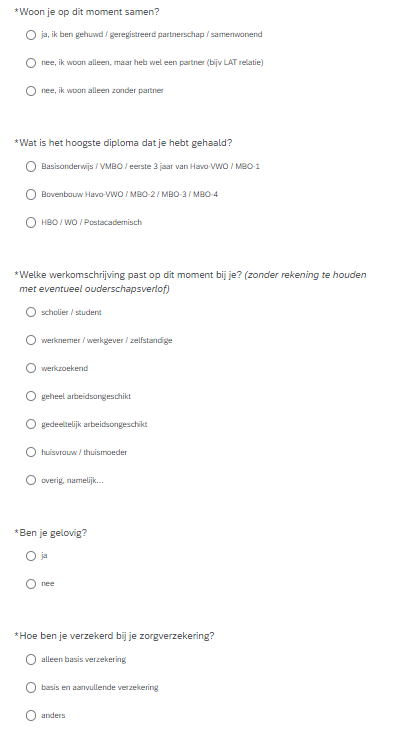
*


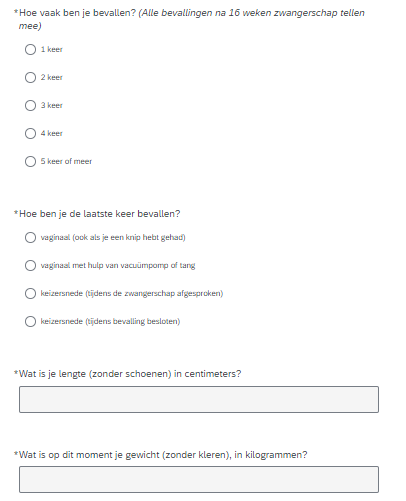


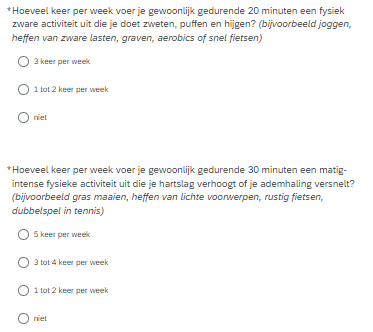


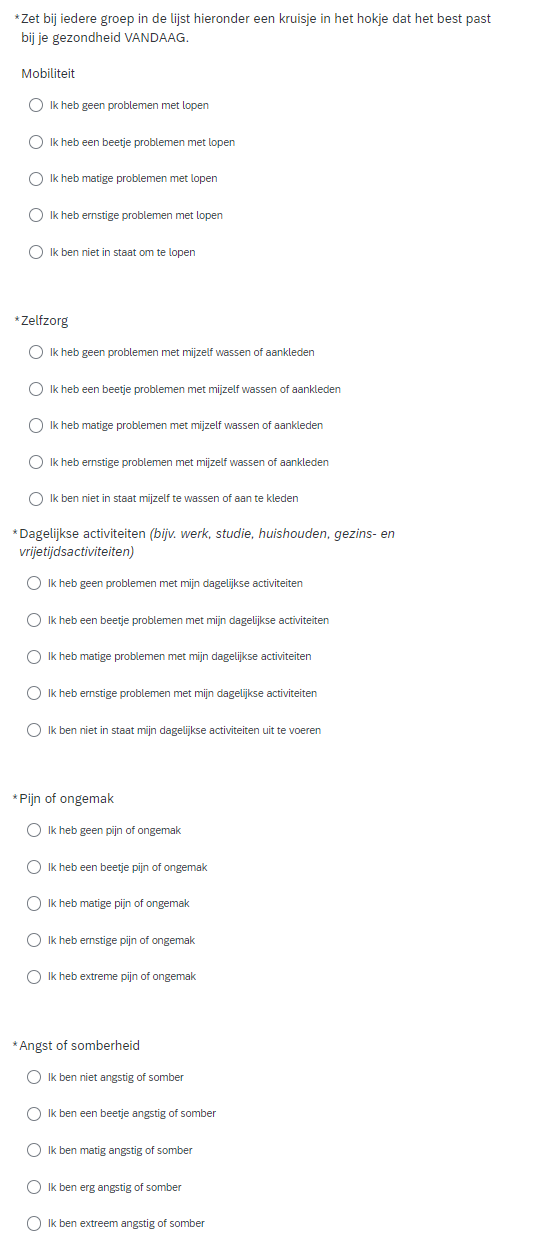


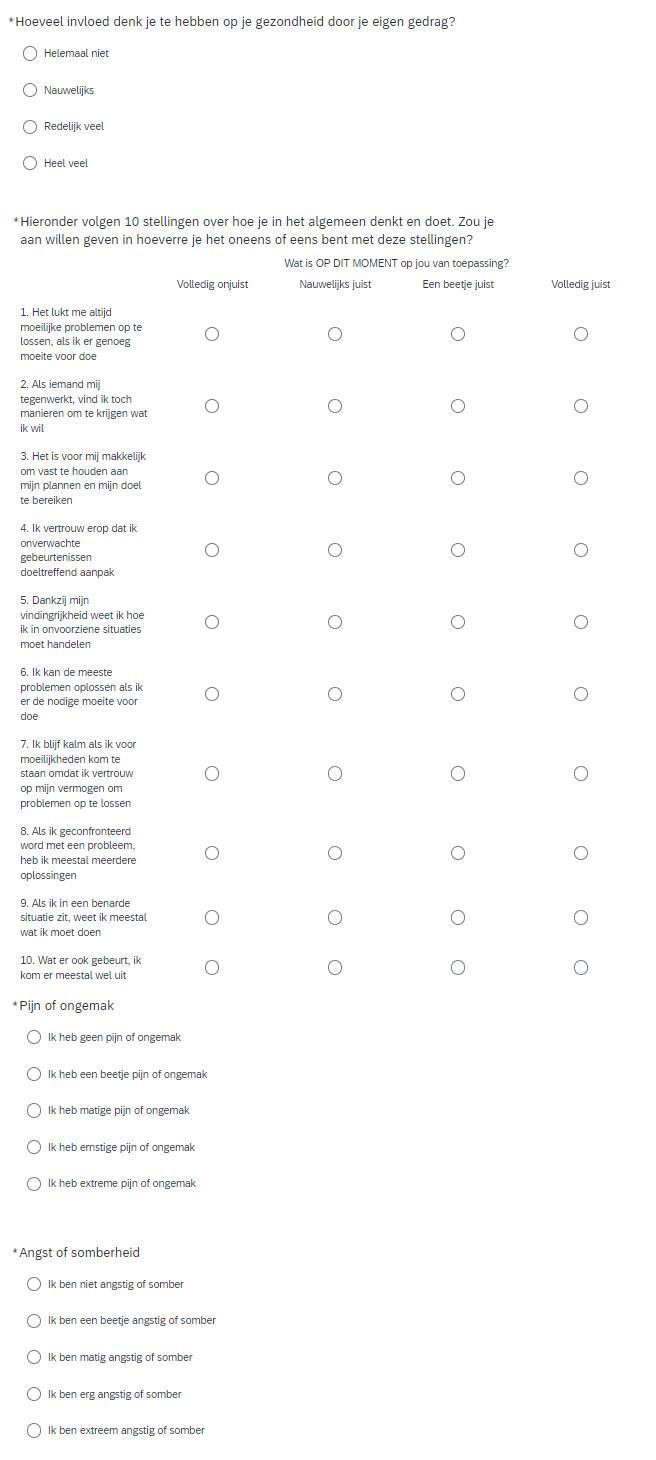


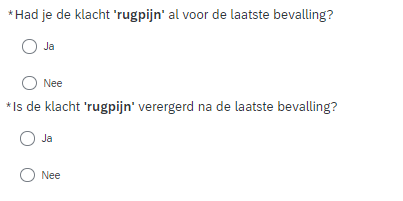

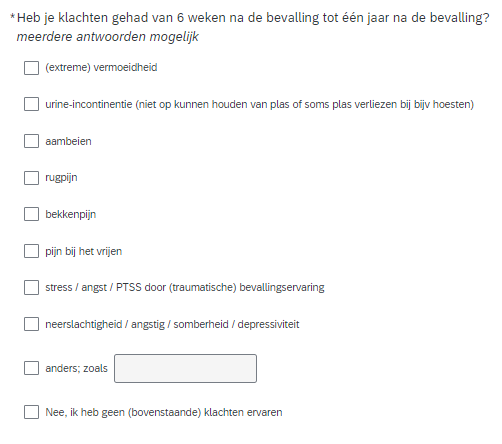

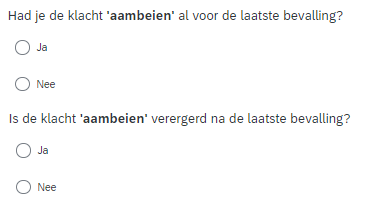

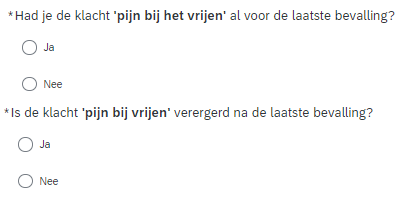

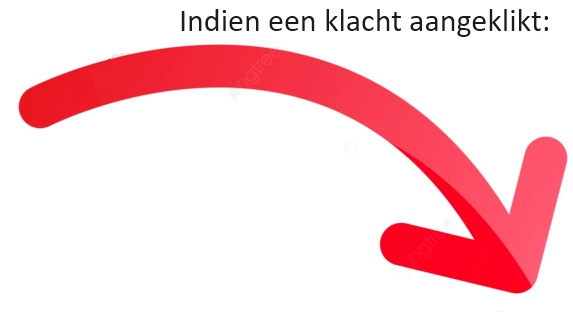


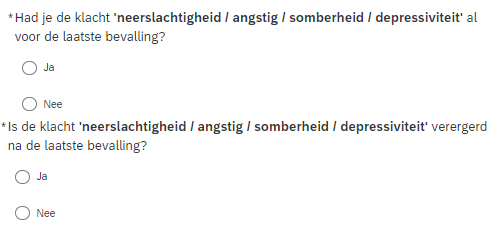


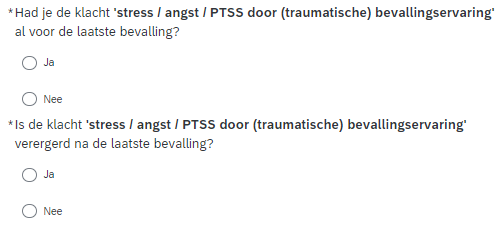

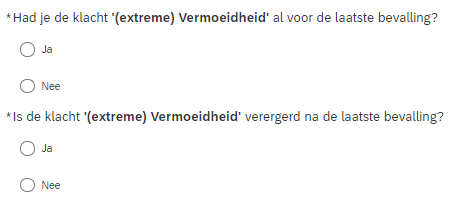

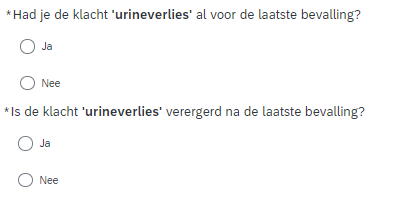

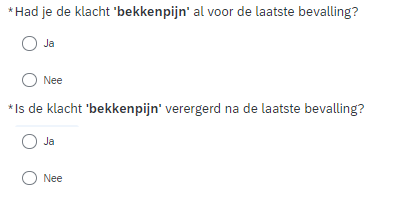


**
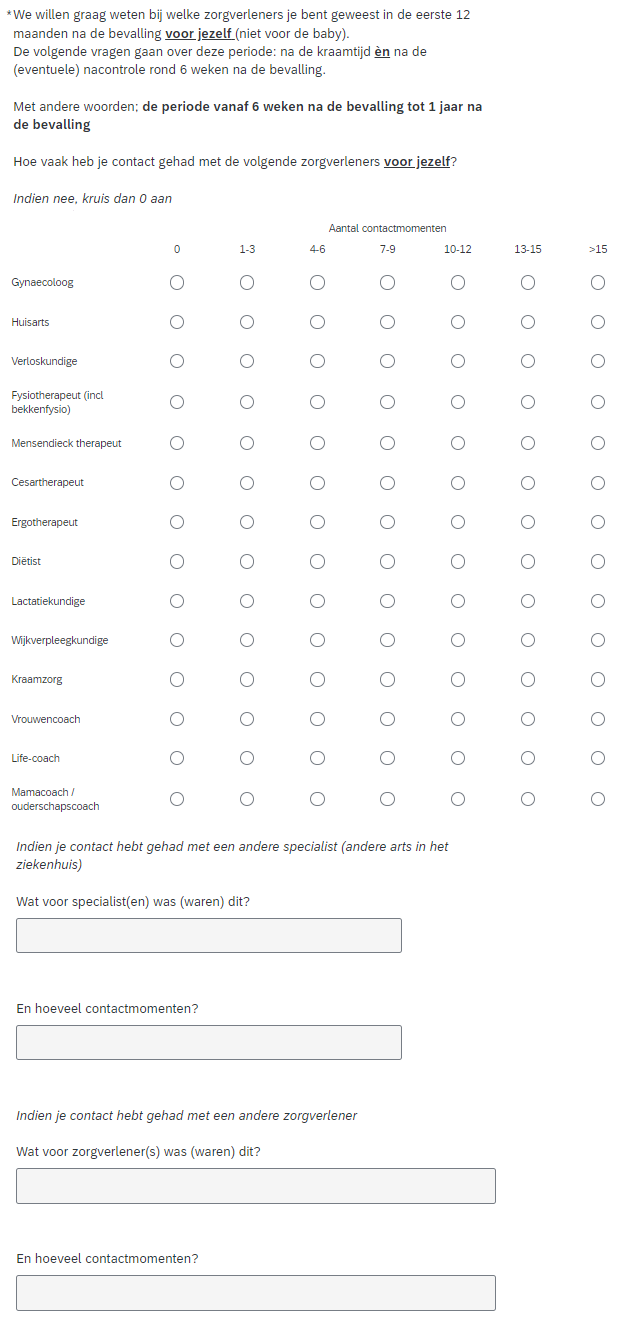
**

# **
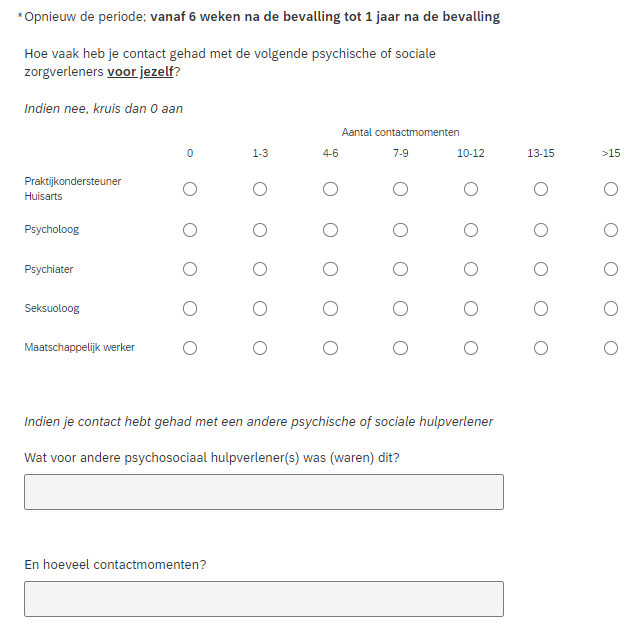
**


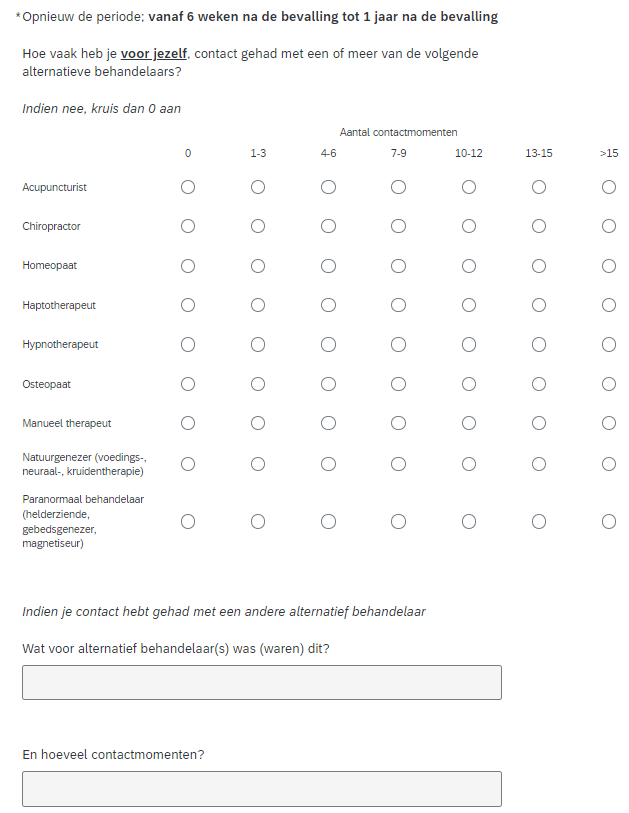


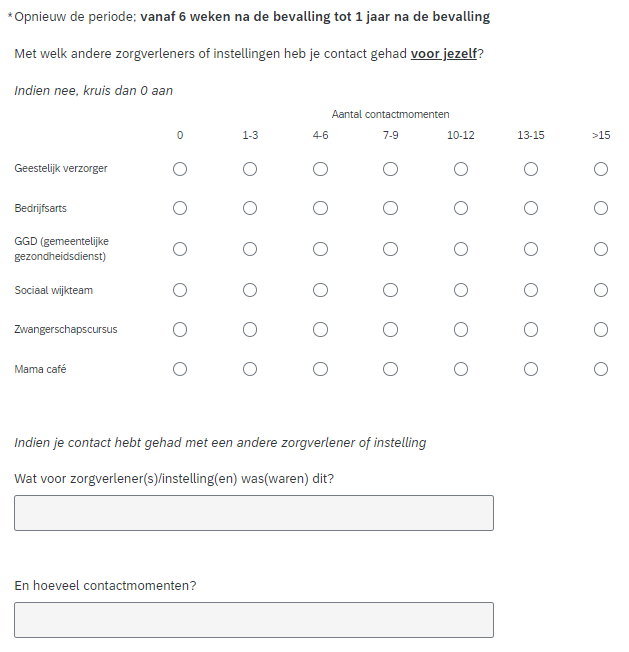


# **
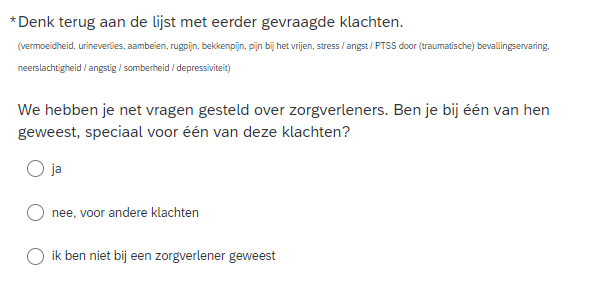
**

# **
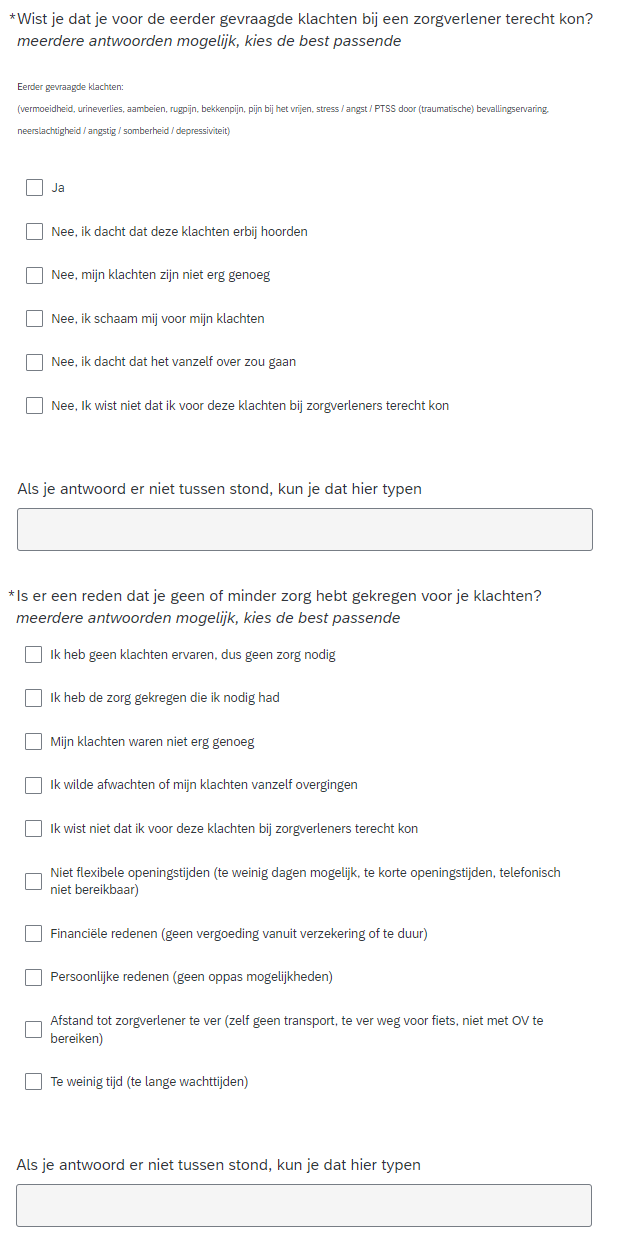
**

**
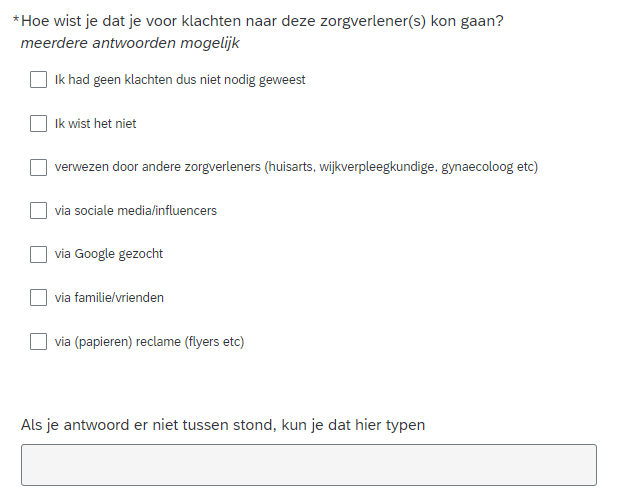
**


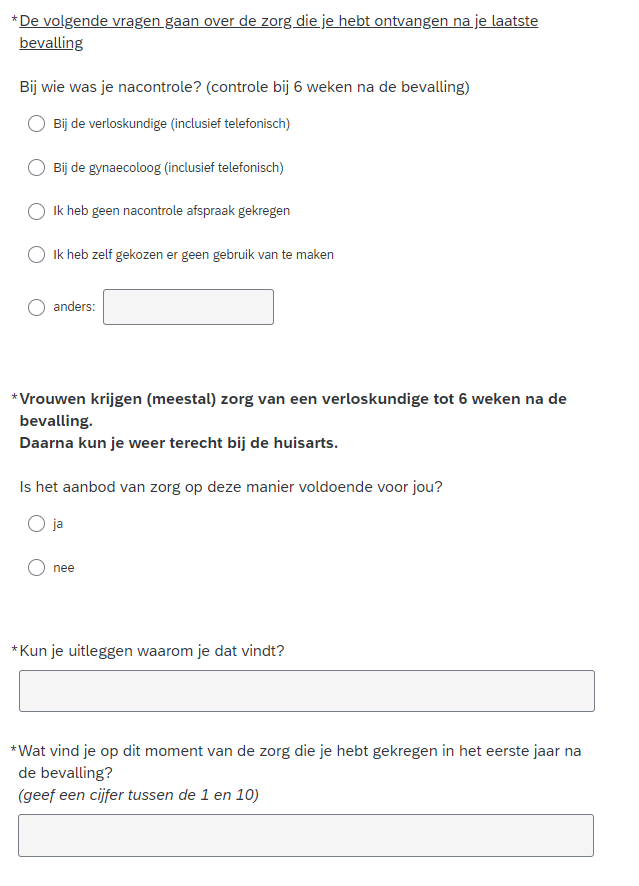


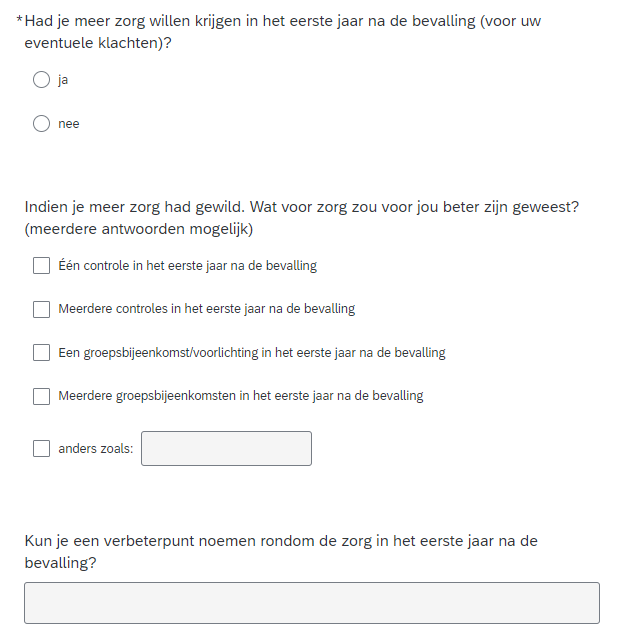


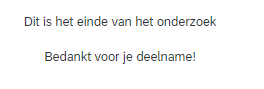

Supplement: Supplementary file 1 — Appendix S1. Dutch Questionnaire [file JMWH-71-113-s007.docx]

# **
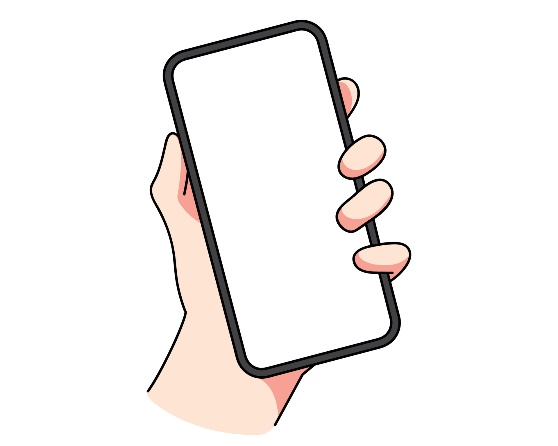
Supplement Information: Q****uestionnaire English**


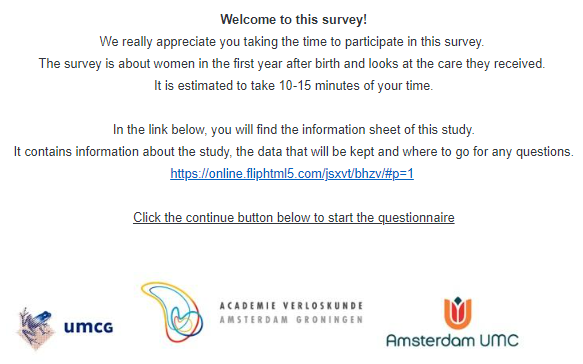


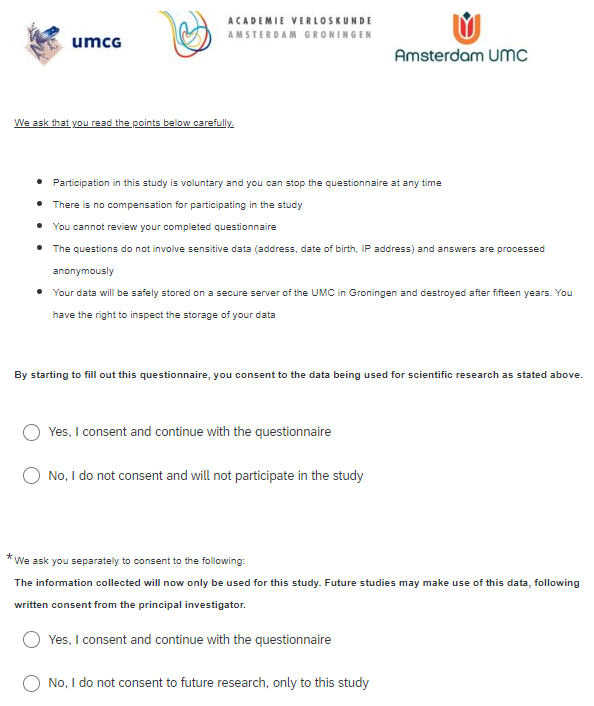


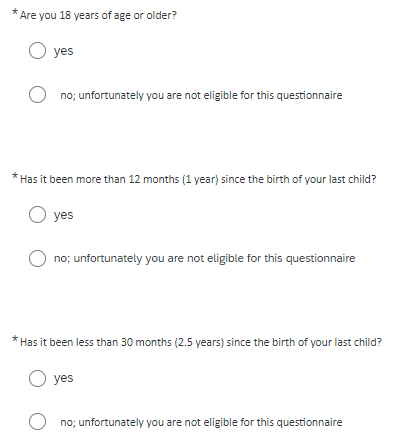


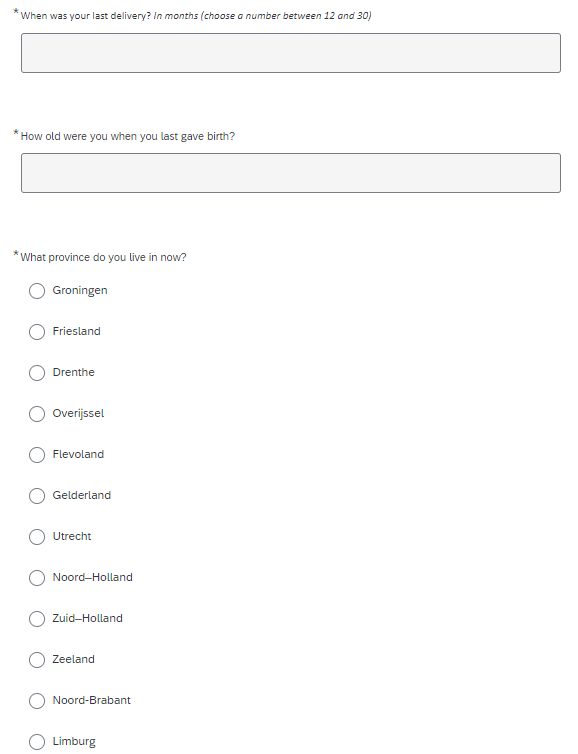


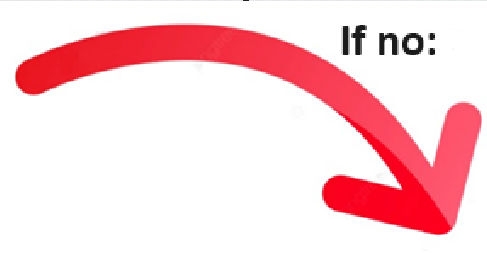

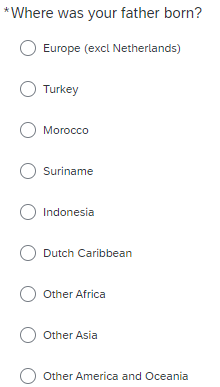

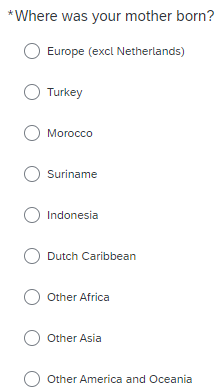

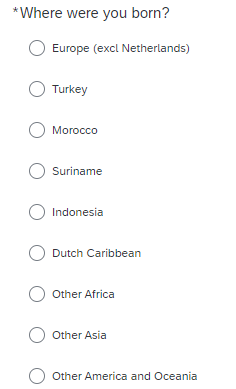

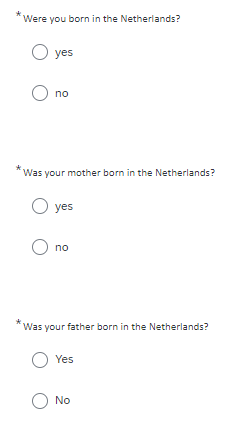


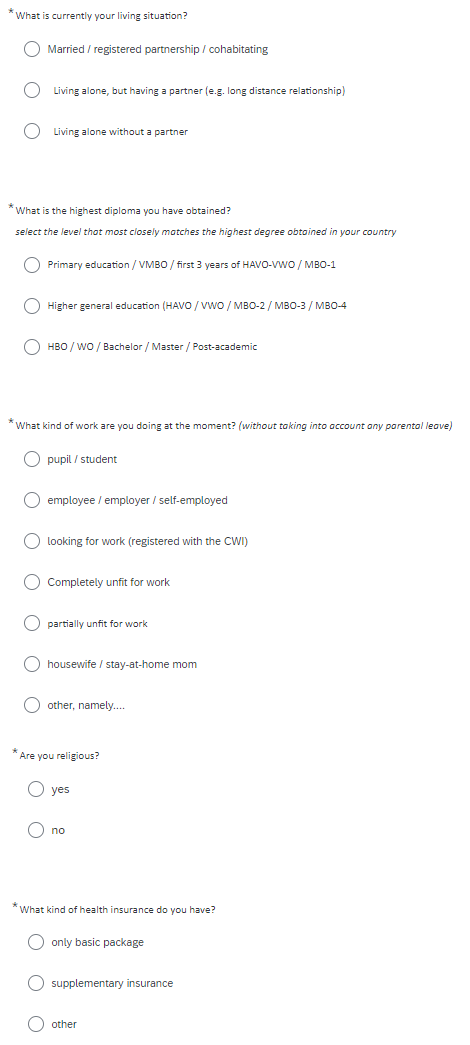


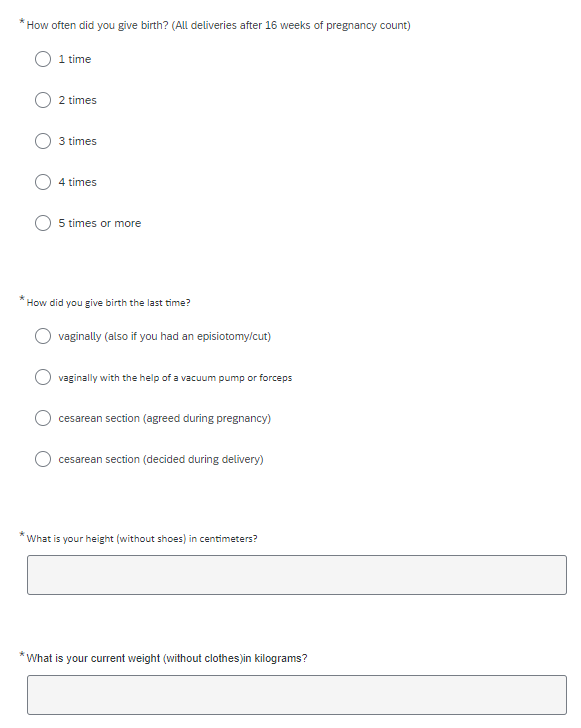


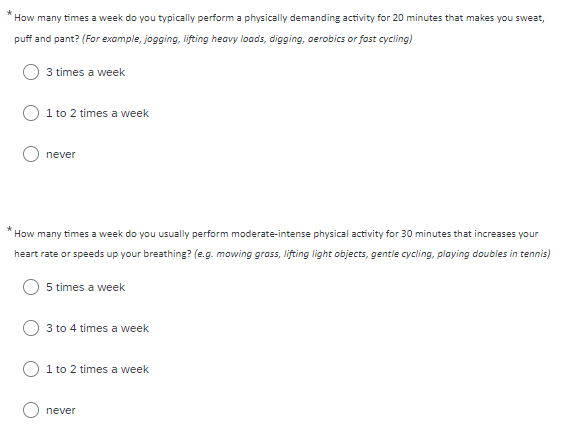


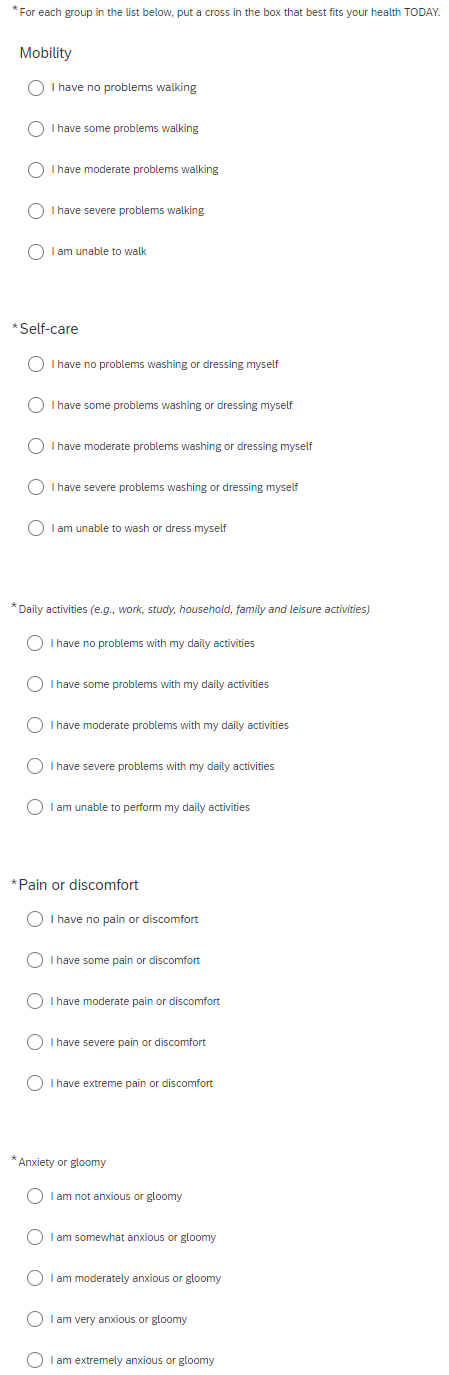


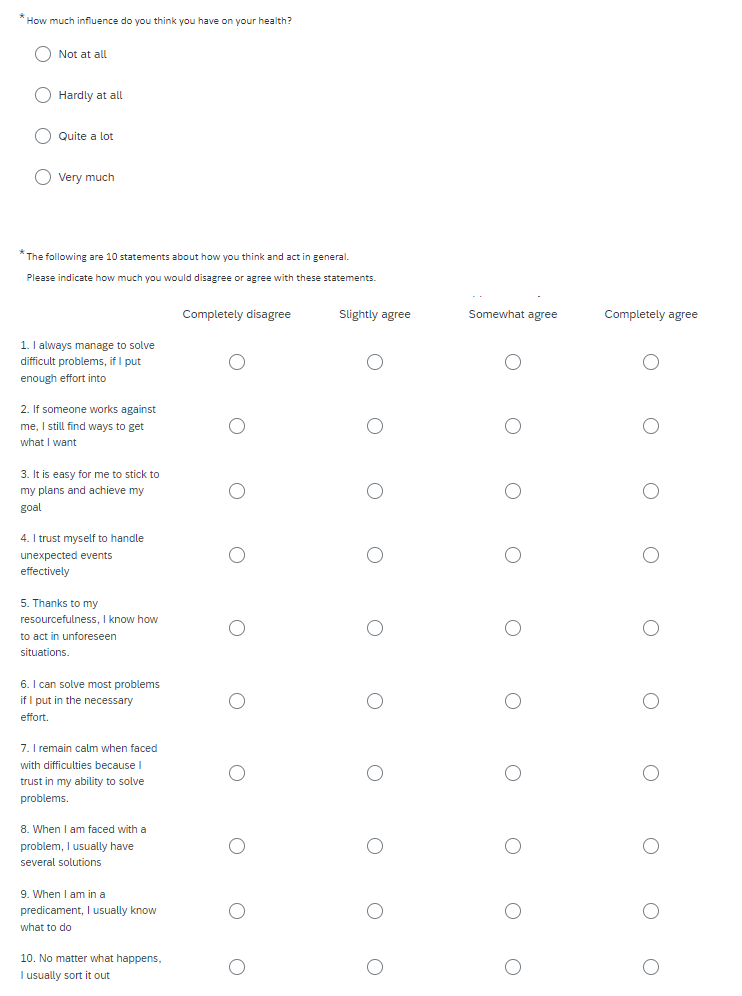


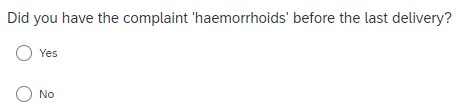

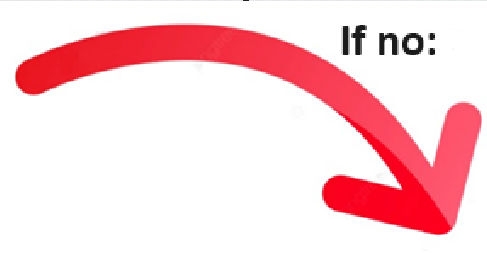

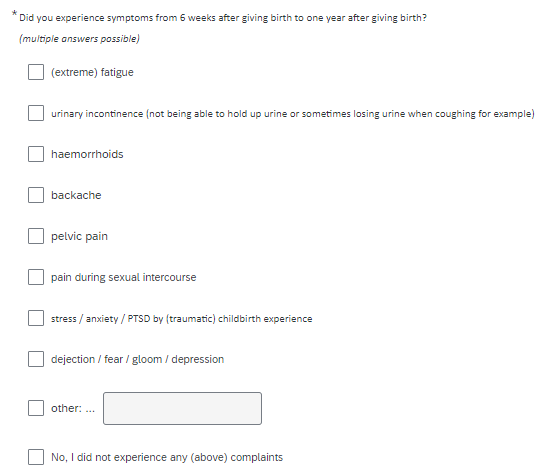


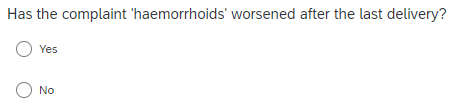


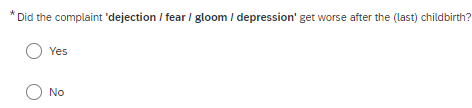

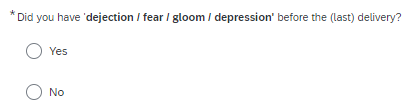


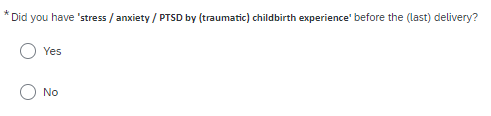


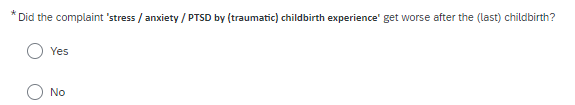


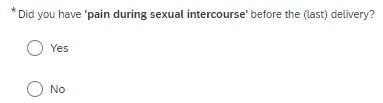


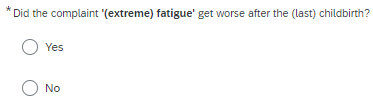


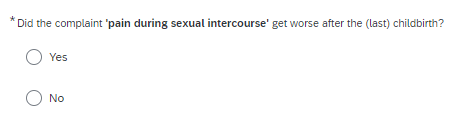


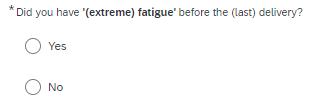


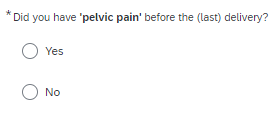

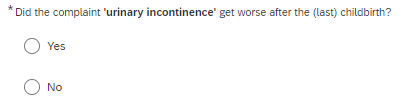


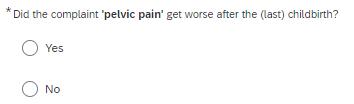

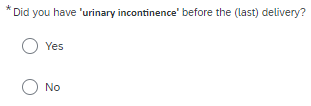


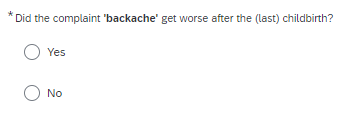

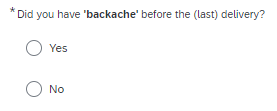


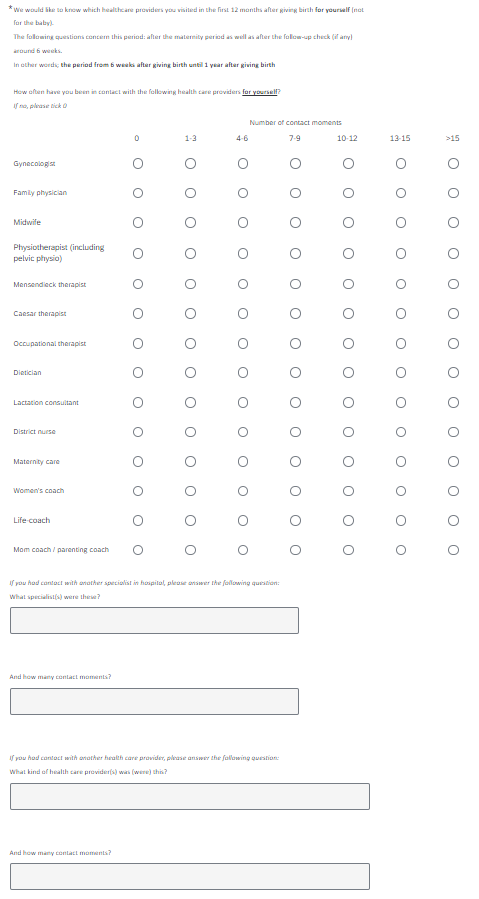


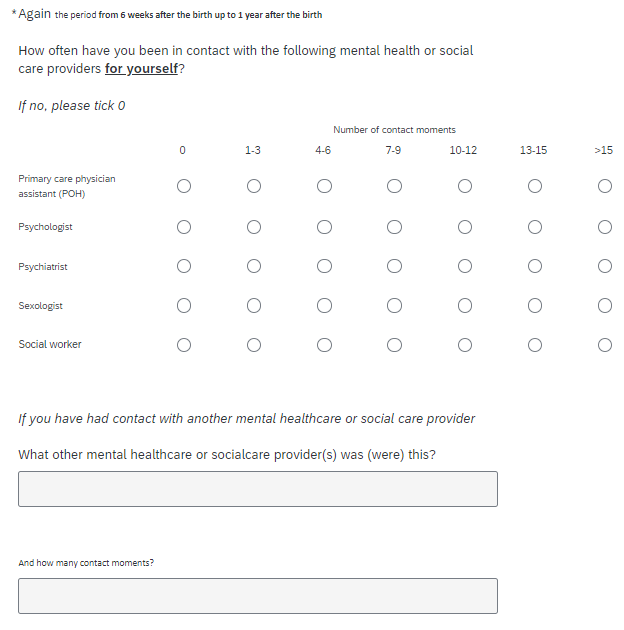


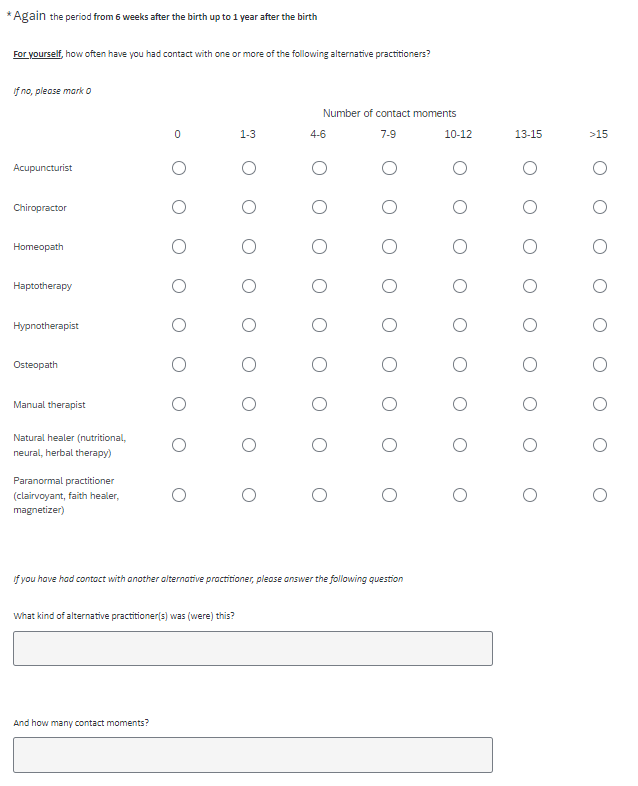


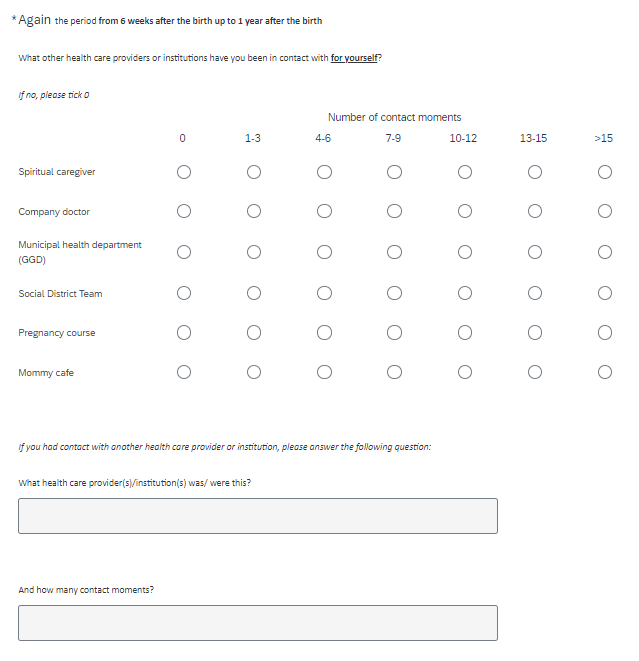


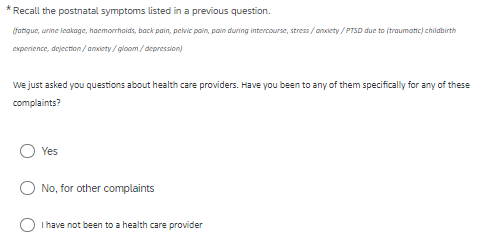


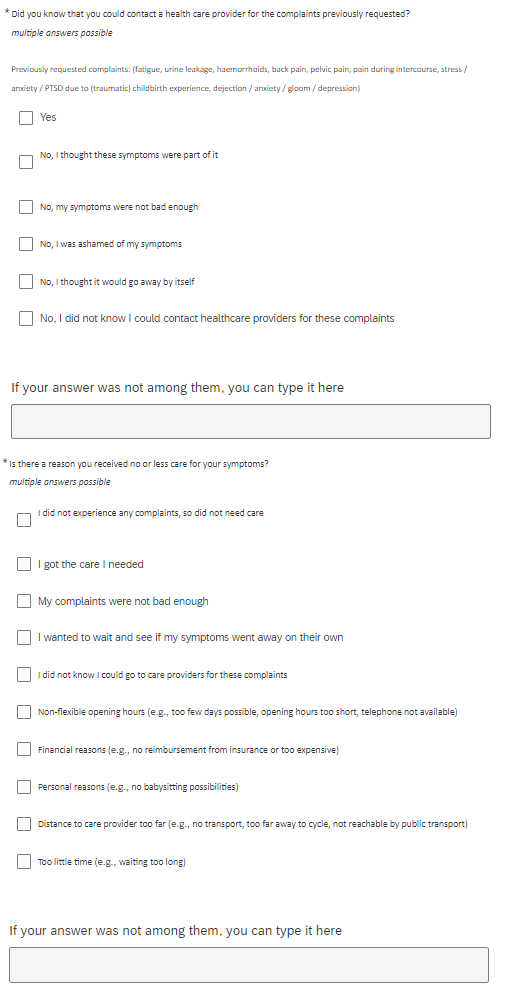


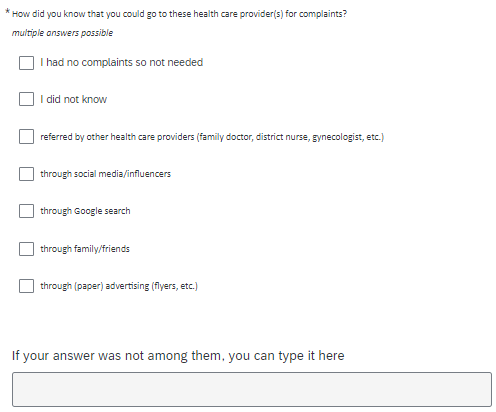


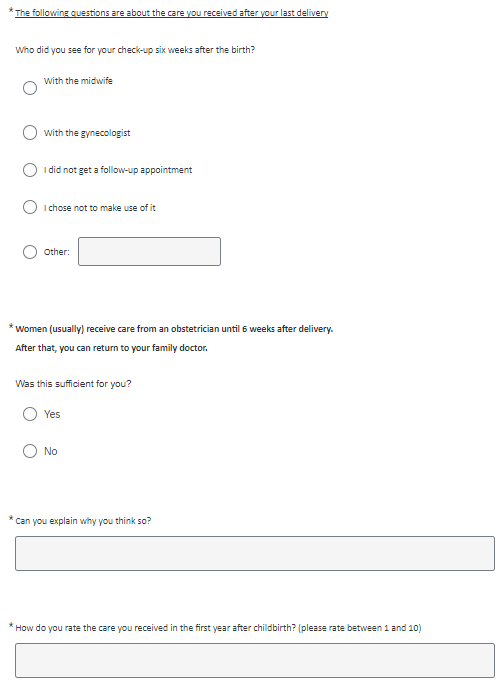


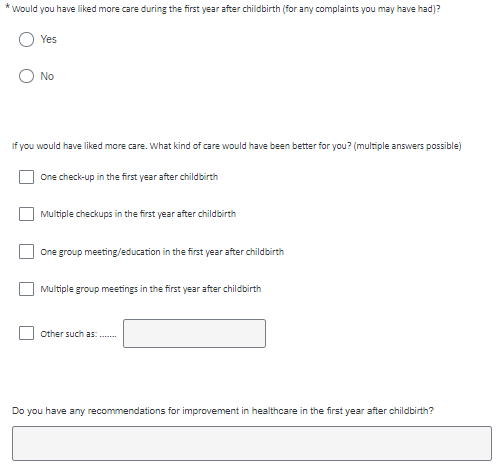


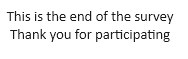

Supplement: Supplementary file 2 — Appendix S2. English Questionnaire [file JMWH-71-113-s008.docx]

## Slide 1
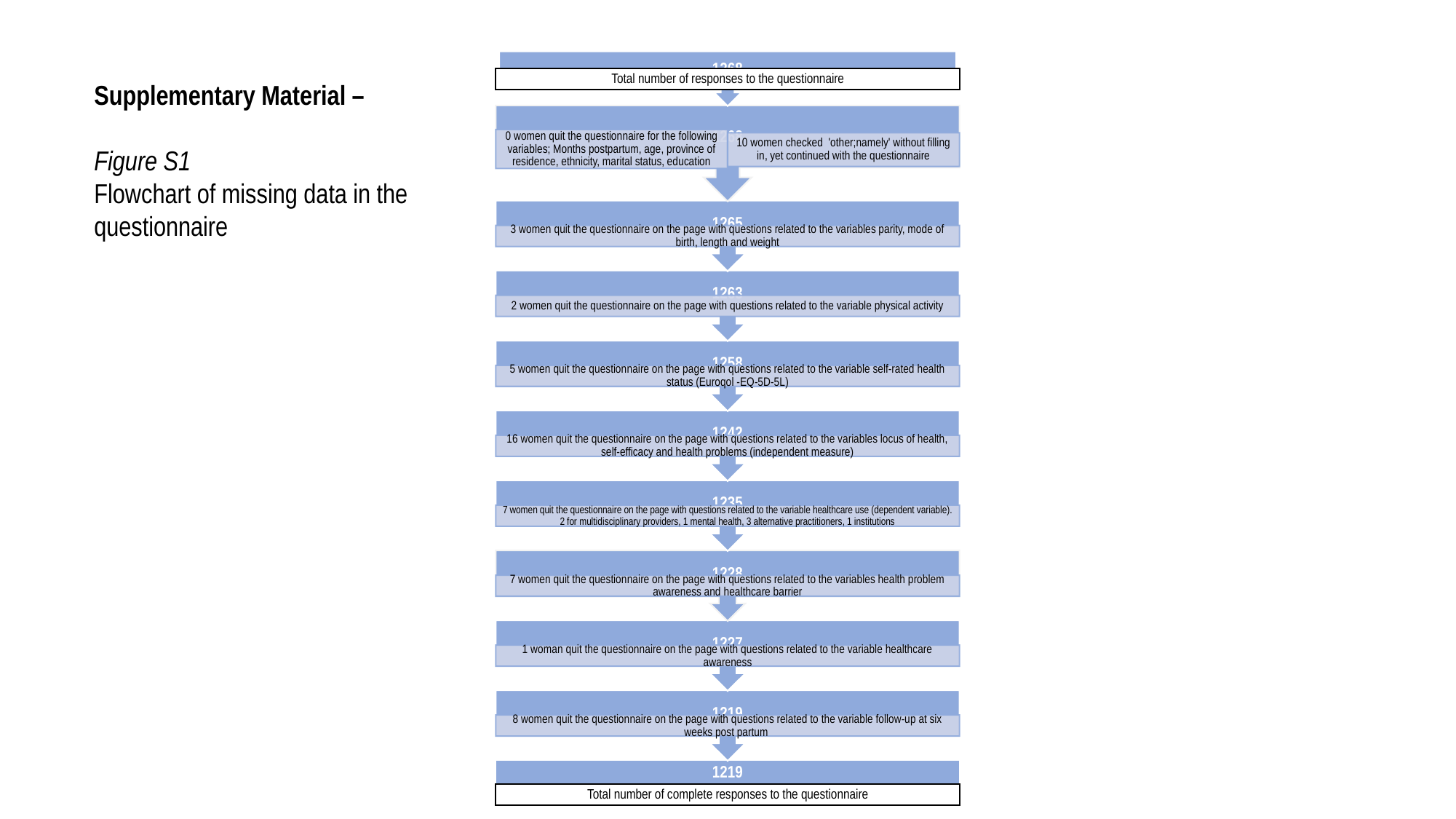

Supplementary Material –
Figure S1
Flowchart of missing data in the questionnaire

Supplement: Supplementary file 5 — Figure S2. Flowchart of Missing Data in the Questionnaire [file JMWH-71-113-s006.pptx]
