# Supplementary material for: Prevalence and Determinants of Health Care Utilization Among Dutch Women in the First Year Postpartum
Source: J Midwifery Womens Health. 2025 Dec 4;71(1):113–25. doi: 10.1111/jmwh.70055 (PMC12914622; doi:10.1111/jmwh.70055)
Supplement: Supplementary file 4 — Figure S1. Recruitment Advertising Online and Flyer [file JMWH-71-113-s005.pptx]

## Slide 1
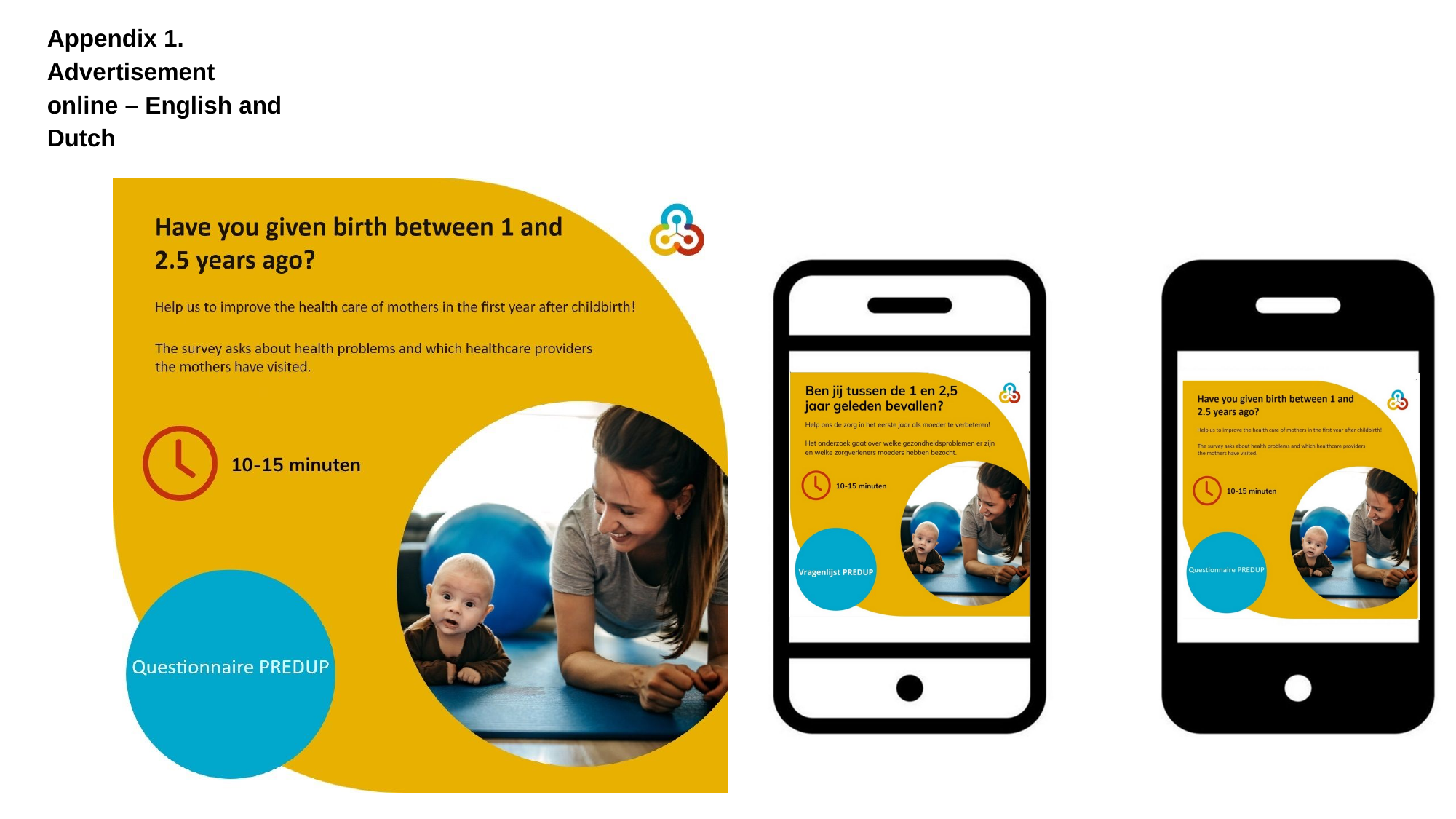

Appendix 1. Advertisement online – English and Dutch

## Slide 2
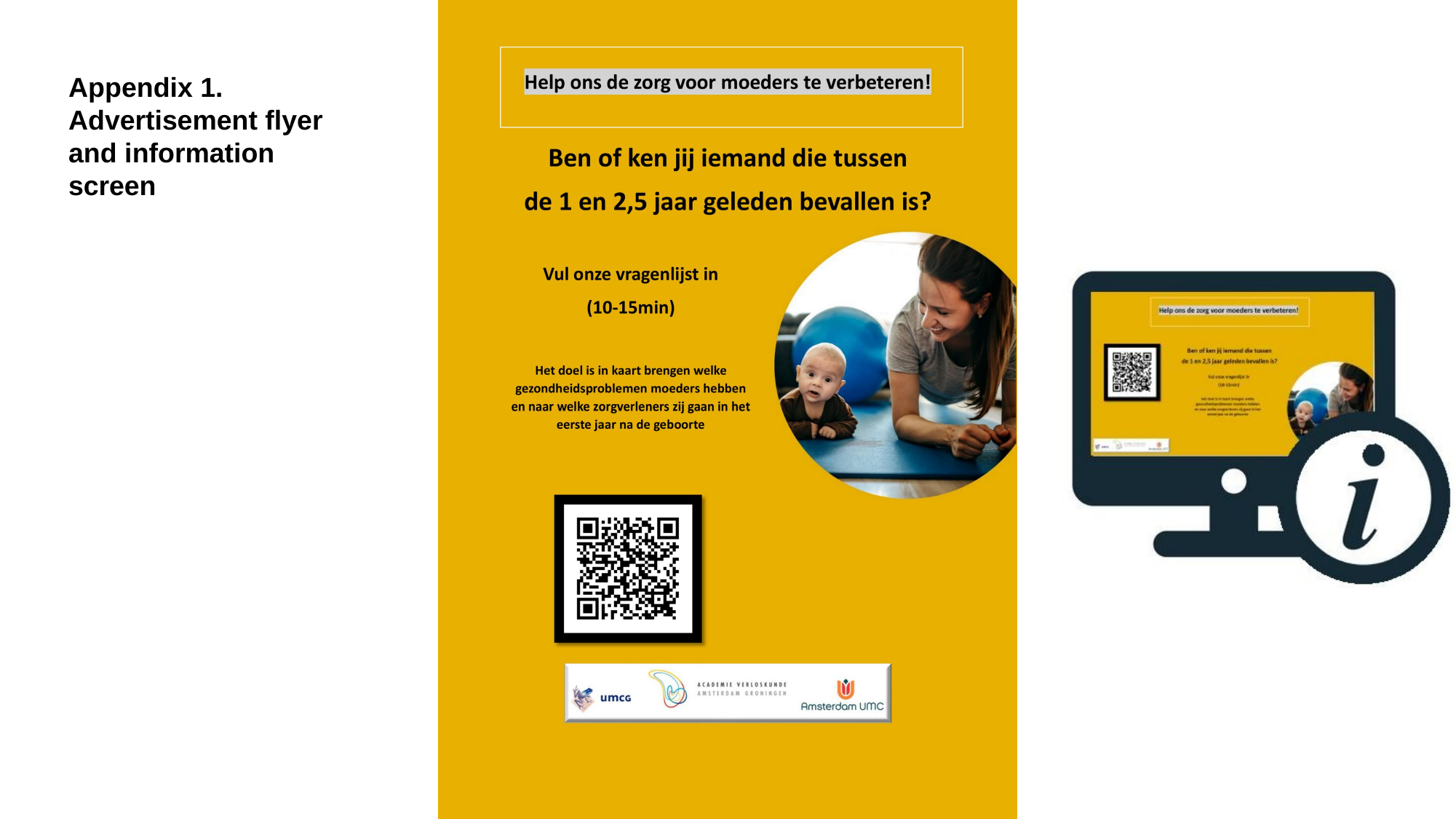

Appendix 1. Advertisement flyer and information screen
