# Supplementary material for: Prevalence and Determinants of Health Care Utilization Among Dutch Women in the First Year Postpartum
Source: J Midwifery Womens Health. 2025 Dec 4;71(1):113–25. doi: 10.1111/jmwh.70055 (PMC12914622; doi:10.1111/jmwh.70055)

Supporting Information:

Figure S3: Distribution of health care utilization among women in the period 6 weeks to 12 months postpartum per type of health care provider in percentages

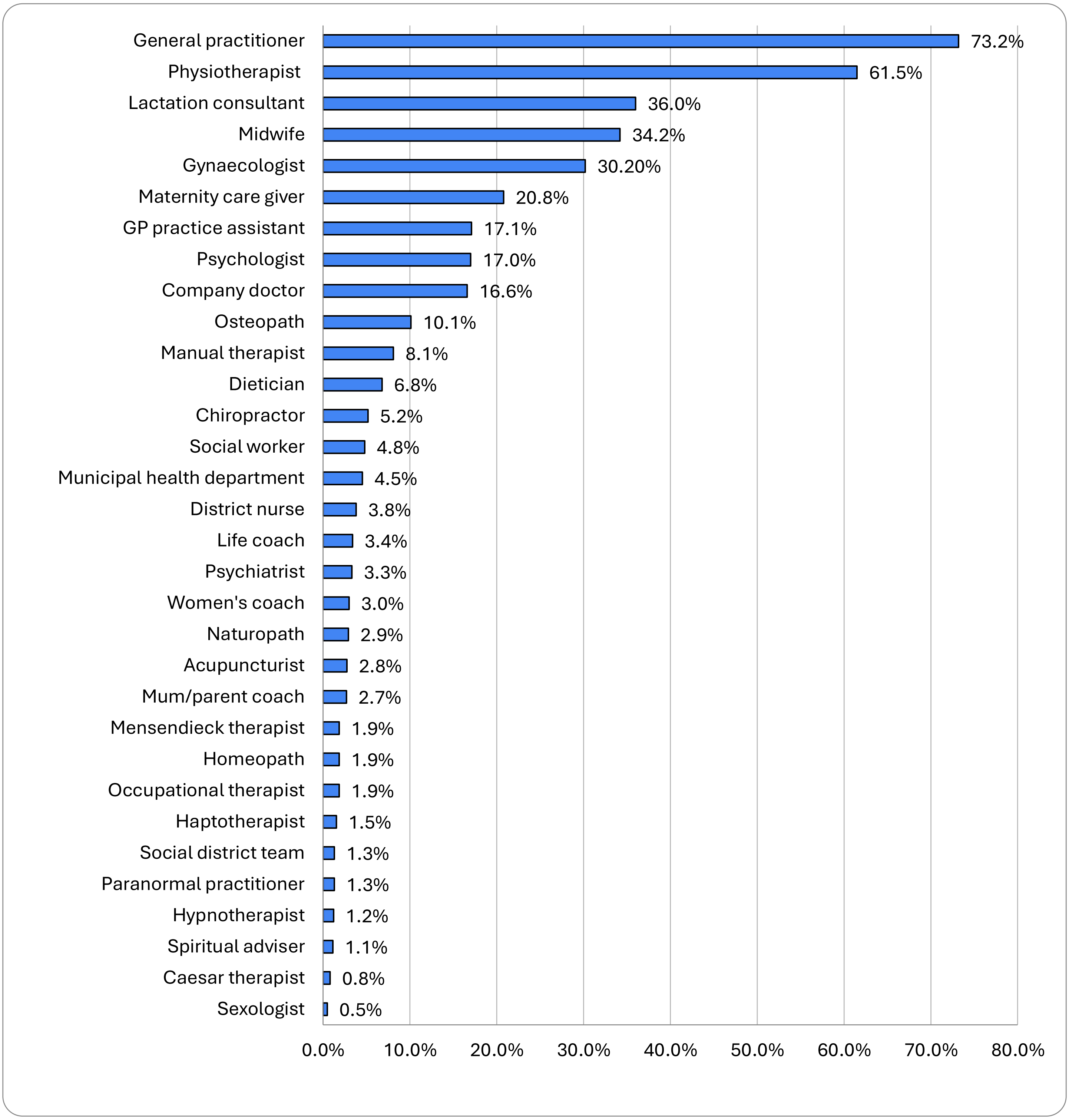

Supplement: Supplementary file 6 — Figure S3. Distribution of Health Care Utilization Among Women in the Period 6 Weeks To 12 Months Postpartum, per Type of Health Care Provider, in Percentages [file JMWH-71-113-s009.pdf]
