# Supplementary material for: Prevalence and Determinants of Health Care Utilization Among Dutch Women in the First Year Postpartum
Source: J Midwifery Womens Health. 2025 Dec 4;71(1):113–25. doi: 10.1111/jmwh.70055 (PMC12914622; doi:10.1111/jmwh.70055)
Supplement: Supplementary file 7 — Table S1. Measurement and Categorization of Questionnaire Variables [file JMWH-71-113-s001.docx]

Table S1. Measurement and Categorization of Questionnaire Variables

| **Variable** | **Questionnaire item and response options** | **Measurement scale** | **Categorization** |
| --- | --- | --- | --- |
| Age | 1 item – open response | Continuous | Transformed into a three-level categorical variable according to the CBS classifications.^1^  (1) ≤20 years (2) 21-34 years (3) ≥35 years |
| Ethnicity | 3 items – 2 response options  (respondent born in the Netherlands, mother born in the Netherlands, father born in the Netherlands) | Nominal | An aggregated variable was formed by combining responses (yes or no) from three items.  1. Dutch origin; respondent and both parents born in the Netherlands  2. Child of migrant: respondents born in the Netherlands with one or two parents born abroad  3. Migrant: foreign-born respondents  Categorized according CBS classifications.^2^ |
| Relationship status | 1 item – 3 response options | Nominal | Responses were grouped into two categories:  1. married or registered partnership or living together  2. (living alone / no relationship) |
| Education level | 1 item – 3 response options | Ordinal | Category division is based on CBS.^3,4^  *1. Low* (primary education, pre-vocational secondary education [VMBO], the first three years of senior general secondary education [HAVO/VWO], or entry-level vocational training [MBO-1]);  *2. Middle* (upper years of senior general secondary education [HAVO/VWO], or intermediate vocational training levels [MBO-2 to MBO-4])  *3. High* (higher professional education [HBO], university education [WO], or post-academic education). |
| Occupational status | 1 item – 7 response options | Ordinal | Responses were grouped into 3 categories, according to the CBS classifications.^2,4^  1. Employed - (student, employed)  2. Unemployed - (job seeking, housewife)  3. Disabled - (Completely unfit for work, partially unfit for work)  Category ‘other, namely’ was excluded as missing. |
| Religion | 1 item – 2 response options | Nominal | Dichotomous variable: (1) yes (2) no |
| Health insurance | 1 item – 2 response options | Nominal | Dichotomous variable: (1) basic health insurance (2) supplementary health insurance |
| Healthcare barrier | 1 item – 9 response options | Nominal | Responses were grouped into 2 categories:  1. 'yes' (personal reasons, proximity of health facility, lack of time, lack of awareness)  2. 'no' (not needed, adequate care, complaints not severe enough, self-resolving, inflexible opening hours, financial constraints) |
| Healthcare awareness | 1 item – 7 response options | Nominal | Responses were grouped into 2 categories for analysis:  1. 'yes' (through referral, through social media, through Google, through family/friends, through advertising)  2. 'no' (I did not know, not needed). |
| Follow-up | 1 item - 4 response options | Nominal | Responses were grouped into 2 categories: (1) ‘yes’ (midwife / gynaecologist)  (2) ‘no’ (own choice / not received) |
| Parity | 1 item – 5 response options | Nominal | Responses were grouped into 2 categories: (1) Primiparous (gave birth 1 time)  (2) Multiparous (gave birth 2 / 3 / 4 / 5 times) |
| Mode of birth | 1 item – 4 response options | Nominal | Responses were grouped into 3 categories:  1. Vaginal birth  2. Assisted vaginal birth  3. Caesarean birth (planned / unplanned) |
| Self-rated health status | 1 item – 5 dimensions - 5 response options  EuroQol (EQ-5D-5L) | Ordinal | Measured by EuroQol (EQ-5D-5L)^a^ The respondent was asked to consider and rate her current state of health in 5 dimensions: mobility, self-care, usual activities, pain/discomfort, and anxiety/depression.^5^ Responses to questions on each dimension could take 1 of 5 values covering 5 levels of severity (no problems/slight problems/moderate problems/severe problems/extreme problems).^5^ Categorized dichotomous in (1) 'no problems' in all dimensions or (2) 'any problems' in levels 2-5 of the dimensions. |
| Health problems | 1 item – 9 response options | Nominal | Responses were grouped into 2 categories:  1. yes ((extreme) fatigue, UI, hemorrhoids, back pain, pelvic pain, dyspareunia, stress/anxiety/posttraumatic stress disorder (PTSD) due to (traumatic) childbirth experiences, low mood/anxiety/glooming/depression)  2. no problems |
| Awareness of health problems | 1 item - | Nominal | Responses were grouped into 2 categories:  1. 'yes'  2. 'no' (perceived as normal, not severe enough, embarrassed, self-resolved, didn't know). |
| BMI | 2 item – open response | Continuous | BMI was calculated from the respondent's current weight (kg) and height (cm) using the Quetelet Index and classified according to the World Health Organization’s classification.^6^ |
| Physical Activity | 2 item – 3 and 4 response options | Nominal | The 'Physical Activity Questionnaire'^a^ (VFA) comprises 2 questions about physically intensive activities (scored as 3 times per week=4, 1-2 times per week=2, never=0) and moderately intensive activities (scored as 5 times per week=4, 3-4 times per week=2, 1-2 times per week=1, never=0).^7^ The VFA score is the sum of both questions, with a range of 0-8, divided into 2 categories: (1) sufficiently active (≥4) o(2) insufficiently active (≤3).^7^ |
| Health of Locus | 1 item – 4 response options | Nominal | Responses for health locus of control (HLOC) were grouped into 2 categories:  (1) ‘no’ (not at all, hardly at all) (2) ‘yes’ (quite a lot, very much) |
| Self-efficacy | 1 item – 10 statements – 4 point scale | Ordinal | The GSES assessed how a person generally copes with stressors or difficult situations in life. Ten statements were rated on a 4-point scale (completely disagree=1, slightly agree=2, somewhat agree=3, and completely agree=4).^8^ The GSES scale ranges from 10 to 40, with higher scores indicating greater self-efficacy.^8^ The mean for the general Dutch population is 32.^9^ |

Abbreviation: classification of Statistics Netherlands (CBS)

^a^ Validated measurement instrument

References of Table S1.

1. Having Children. Statistics Netherlands website. Accessed 10th February 2024. <https://www.cbs.nl/nl-nl/visualisaties/dashboard-bevolking/levensloop/kinderen-krijgen#:~:text=In%201975%20was%20van%2090,van%2035%20jaar%20of%20ouder>.

2. Introducing new population classification by origin. Statistics Netherlands website. Accessed 16th May 2024. <https://www.cbs.nl/en-gb/news/2022/07/cbs-introducing-new-population-classification-by-origin>

3. Standard Education Classification (Dutch: Standaard Onderwijsindeling) 2021. CBS SN- <https://www-cbs-nl.proxy-ub.rug.nl/nl-nl/onze-diensten/methoden/classificaties/onderwijs-en-beroepen/standaard-onderwijsindeling--soi--/standaard-onderwijsindeling-2021>

4. Onderwijsniveau van moeders van kinderen van 0-18 jaar. Statistics Netherlands website. Accessed 16th May, 2024. <https://www.cbs.nl/nl-nl/maatwerk/2021/51/onderwijsniveau-van-moeders-van-kinderen-van-0-18-jaar>

5. Long D, Polinder S, Bonsel GJ, Haagsma JA. Test–retest reliability of the EQ-5D-5L and the reworded QOLIBRI-OS in the general population of Italy, the Netherlands, and the United Kingdom. *Qual Life Res*. 2021;30(10):2961-2971. doi:10.1007/s11136-021-02893-3

6. Nutrition Landscape Information System (NLiS). Malnutrition in women. World Health Organization (WHO) website. Accessed 15th October 2023. <https://www.who.int/data/nutrition/nlis/info/malnutrition-in-women#:~:text=BMI%20%3C18.5%3A%20underweight,BMI%20%E2%89%A530.0%3A%20obesity>

7. Prochaska JJ, Sallis JF, Long B. A physical activity screening measure for use with adolescents in primary care. *Arch Pediatr Adolesc Med*. 2001;155(5):554-9. doi:10.1001/archpedi.155.5.554

8. Schwarzer R, Born A. Optimistic self-beliefs: Assessment of general perceived self-efficacy in thirteen cultures. *World Psychol*. 1997;3:177-190.

9. Scholz U, Gutiérrez-Doña B, Sud S, Schwarzer R. Is General Self-Efficacy a Universal Construct? Psychometric Findings from 25 Countries. *Eur J Psychol Assess*. 2002;18:242-251. doi:10.1027//1015-5759.18.3.242
