# Supplementary material for: Prevalence and Determinants of Health Care Utilization Among Dutch Women in the First Year Postpartum
Source: J Midwifery Womens Health. 2025 Dec 4;71(1):113–25. doi: 10.1111/jmwh.70055 (PMC12914622; doi:10.1111/jmwh.70055)
Supplement: Supplementary file 8 — Table S2. Distribution of Health Problems Reported by Respondents in the Open Text Box [file JMWH-71-113-s002.pptx]

## Slide 1
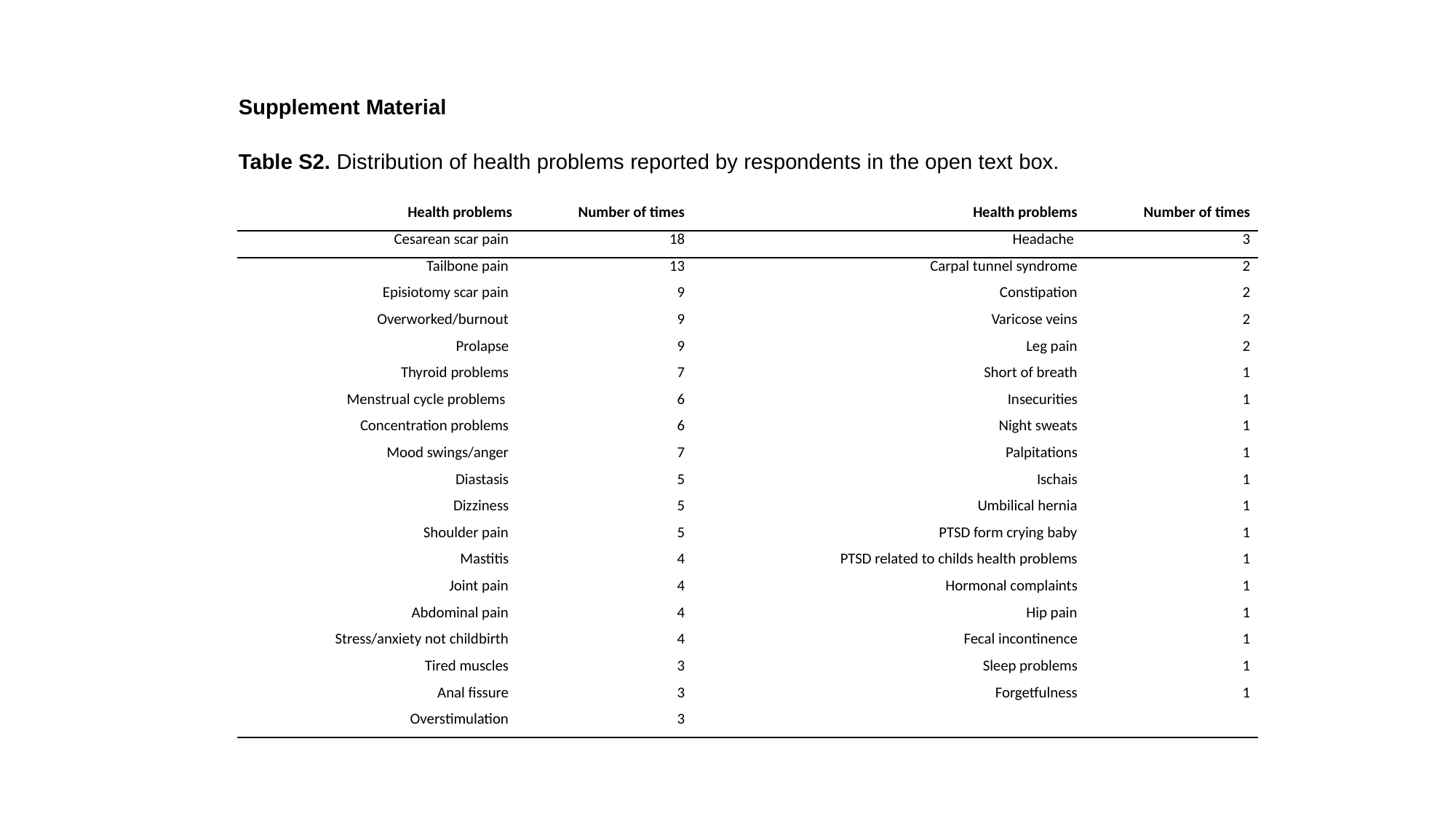

Supplement Material
Table S2. Distribution of health problems reported by respondents in the open text box.
| Health problems | Number of times | Health problems | Number of times |
| --- | --- | --- | --- |
| Cesarean scar pain | 18 | Headache | 3 |
| Tailbone pain | 13 | Carpal tunnel syndrome | 2 |
| Episiotomy scar pain | 9 | Constipation | 2 |
| Overworked/burnout | 9 | Varicose veins | 2 |
| Prolapse | 9 | Leg pain | 2 |
| Thyroid problems | 7 | Short of breath | 1 |
| Menstrual cycle problems | 6 | Insecurities | 1 |
| Concentration problems | 6 | Night sweats | 1 |
| Mood swings/anger | 7 | Palpitations | 1 |
| Diastasis | 5 | Ischais | 1 |
| Dizziness | 5 | Umbilical hernia | 1 |
| Shoulder pain | 5 | PTSD form crying baby | 1 |
| Mastitis | 4 | PTSD related to childs health problems | 1 |
| Joint pain | 4 | Hormonal complaints | 1 |
| Abdominal pain | 4 | Hip pain | 1 |
| Stress/anxiety not childbirth | 4 | Fecal incontinence | 1 |
| Tired muscles | 3 | Sleep problems | 1 |
| Anal fissure | 3 | Forgetfulness | 1 |
| Overstimulation | 3 | | |
