# Supplementary material for: Prevalence and Determinants of Health Care Utilization Among Dutch Women in the First Year Postpartum
Source: J Midwifery Womens Health. 2025 Dec 4;71(1):113–25. doi: 10.1111/jmwh.70055 (PMC12914622; doi:10.1111/jmwh.70055)
Supplement: Supplementary file 9 — Table S3. Distribution of Health Care Providers Reported by Respondents in the Open Text Box [file JMWH-71-113-s004.pptx]

## Slide 1
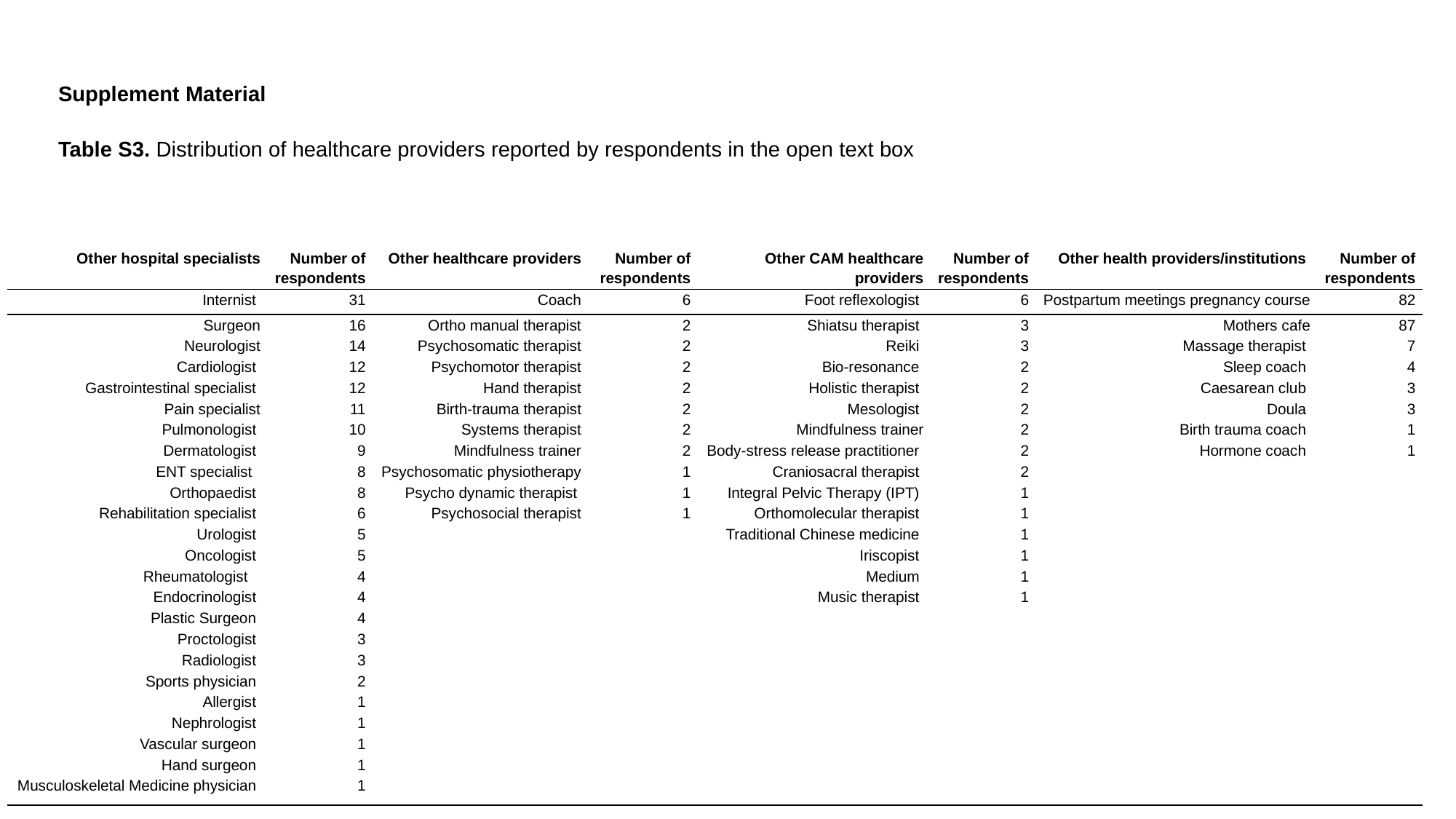

Supplement Material
Table S3. Distribution of healthcare providers reported by respondents in the open text box
| Other hospital specialists | Number of respondents | Other healthcare providers | Number of respondents | Other CAM healthcare providers | Number of respondents | Other health providers/institutions | Number of respondents |
| --- | --- | --- | --- | --- | --- | --- | --- |
| Internist | 31 | Coach | 6 | Foot reflexologist | 6 | Postpartum meetings pregnancy course | 82 |
| Surgeon | 16 | Ortho manual therapist | 2 | Shiatsu therapist | 3 | Mothers cafe | 87 |
| Neurologist | 14 | Psychosomatic therapist | 2 | Reiki | 3 | Massage therapist | 7 |
| Cardiologist | 12 | Psychomotor therapist | 2 | Bio-resonance | 2 | Sleep coach | 4 |
| Gastrointestinal specialist | 12 | Hand therapist | 2 | Holistic therapist | 2 | Caesarean club | 3 |
| Pain specialist | 11 | Birth-trauma therapist | 2 | Mesologist | 2 | Doula | 3 |
| Pulmonologist | 10 | Systems therapist | 2 | Mindfulness trainer | 2 | Birth trauma coach | 1 |
| Dermatologist | 9 | Mindfulness trainer | 2 | Body-stress release practitioner | 2 | Hormone coach | 1 |
| ENT specialist | 8 | Psychosomatic physiotherapy | 1 | Craniosacral therapist | 2 | | |
| Orthopaedist | 8 | Psycho dynamic therapist | 1 | Integral Pelvic Therapy (IPT) | 1 | | |
| Rehabilitation specialist | 6 | Psychosocial therapist | 1 | Orthomolecular therapist | 1 | | |
| Urologist | 5 | | | Traditional Chinese medicine | 1 | | |
| Oncologist | 5 | | | Iriscopist | 1 | | |
| Rheumatologist | 4 | | | Medium | 1 | | |
| Endocrinologist | 4 | | | Music therapist | 1 | | |
| Plastic Surgeon | 4 | | | | | | |
| Proctologist | 3 | | | | | | |
| Radiologist | 3 | | | | | | |
| Sports physician | 2 | | | | | | |
| Allergist | 1 | | | | | | |
| Nephrologist | 1 | | | | | | |
| Vascular surgeon | 1 | | | | | | |
| Hand surgeon | 1 | | | | | | |
| Musculoskeletal Medicine physician | 1 | | | | | | |
